# Survey, taxonomy, and emerging paradigms of societal digital twins for public health preparedness

**DOI:** 10.1038/s41746-025-01737-5

**Published:** 2025-08-13

**Authors:** Muhammad Waqas Rehan, Muhammad Maaz Rehan

**Affiliations:** 1 University of Lübeck, Institute for Software Engineering and Programming Languages (ISP), 23562 Lübeck, Germany; 2 University of Lancashire, School of Engineering and Computing, Department of Computer Science, PR1 2HE Preston, Lancashire UK

**Keywords:** Computer science, Infectious diseases, Society, Information technology, Decision making

## Abstract

The emergence of SARS-CoV-2 (COVID-19) has demonstrated the severe impact of infectious diseases on global society, politics, and economies. To mitigate future pandemics, preemptive measures for effectively managing infection outbreaks are essential. In this context, Societal Digital Twin (SDT) technology offers a promising solution. To the best of our knowledge, this survey is the premier to conceptualize an SDT framework for infection containment under a novel systematic taxonomy. The framework categorizes infection management into five stages, namely infection initiation, spread, control, combat, and recovery. It provides an overview of SDT approaches within each category, discussing their validation strategies, generalizability, and limitations. Additionally, the survey examines applications, data-driven design issues, key components, and limitations of DT technology in healthcare. Finally, it explores key challenges, open research directions, and emerging paradigms to advance DT applications in the healthcare domain, highlighting smart service paradigms such as SDT as a Smart Service (SDTaaSS) and Healthcare Metaverse as a Smart Service (HMaaSS).

## Introduction

COVID-19 outbreak served as a significant moment for digitization^1^ and underscored the importance of digital technologies in pandemic management. The Digital Twin, closely resembling Cyber Physical Systems (CPS), is a digitization technology that is instrumental in realizing the vision of Industry 4.0^2^. The paradigm “Digital Twin” was initially coined by Michael Grieves in 2003 and is pivotal in the product life cycle for promoting cost-effective manufacturing of high-quality products^3^. The paradigm gained prominence when adopted as a long-term strategic vision by NASA and U.S. Air Force^4^. DT technology is expected to become ubiquitous soon and is estimated to reach a market size of $269 billion by 2032^5^.

**Fig. 1:**
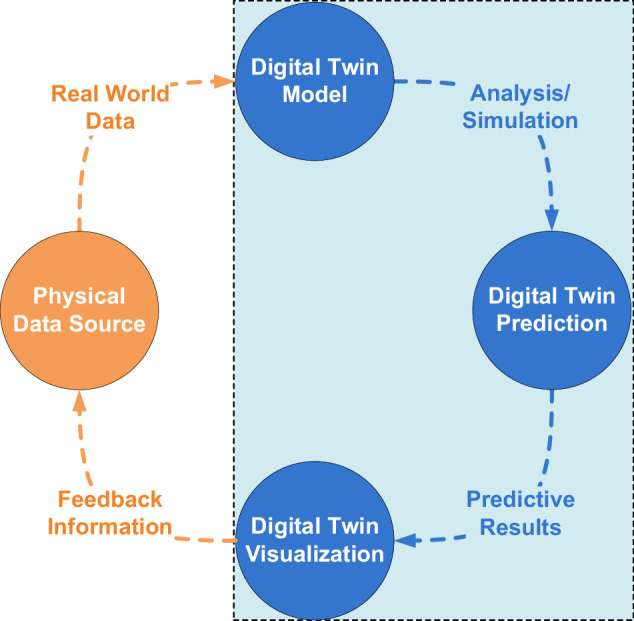
Schematic representation of digital twin life cycle.

Technically speaking, the core of DT embodies a model or blueprint that simulates a physical-world object or system. Data represents a fact, a measurement, or an observation that is fed into the DT framework to replicate or emulate the dynamics of the real-world entity or structure. Consequently, a DT may be characterized as the virtual replica of a living creature, non-living entity^6^, or a digital system from the real-world. Through seamless bidirectional communication with the real-world entity, a DT may continuously collect up-to-date knowledge about the processes and functions of the real-world object. Coupled with built-in intelligence and prediction capabilities of the inherent technologies, a DT may forecast potential issues and send early warnings to the corresponding physical-world entity using a feedback mechanism as shown in Fig. 1. In the healthcare context, maintaining such a close-loop interaction between a DT and a real-world object may ensure the predictive well-being and safety of humans.

DT technology has been serving as a catalyst for revolutionizing healthcare^7^. It has exhibited numerous applications in various health sectors such as neuroscience^8,9^, cardiology^10,11^, diabetes^12^, and so on, thereby playing a significant role in serving humanity. Beyond enhancing human well-being, DT technology has great potential in animal welfare and conservation as well. By facilitating experimentation on digital animal models, DT technology may save around 200 million animals per year^13^, enabling health-related institutions to allocate budget to other significant areas of healthcare.

The recent technological advancements in sensors, the Internet of Things (IoT)—the online connectivity of smart devices with the capability to sense, process and share data over the internet^14^, the Internet of Everything (IoE)—the online networking of data, process, people and things^15^, Automation of Everything (AoE) – the autonomous execution of basic processes without human intervention or external control^16^, Virtual Reality (VR)—creating a simulated three-dimensional (3D) environment, observable using 3D displays and allowing pose tracking to provide an immersive experience^17^, Augmented Reality (AR)—combining real-world surroundings with computer-generated 3D models to enable an interactive experience^18^, Mixed Reality (MR)—blending real-world environments with the simulated virtual world, allowing both to coexist and interact in real time^19^, data-driven analytics, intelligent & automated learning systems, and cloud computing are continuously expanding the capabilities and scope of DT technology in healthcare.

These advancements have further matured DT technology, enabling it to play an increasingly intelligent and pivotal role in personalized and societal healthcare. For example, a DT may facilitate personalized treatment by offering real-time patient monitoring, optimized medical care or surgical planning, proactive disease forecasting, and furnishing tele-medicine & consultation services by considering customized patient models. Likewise, medical training, virtual disease treatment, drug discovery & development, device engineering & testing, hospital and clinical processes optimization are some of the relevant areas for supporting personalized healthcare through DT technology.

The *societal aspects* of DT technology are evident from the numerous approaches targeted for the well-being of the community at large, such as predicting and combating infectious diseases. Although this survey emphasizes emergency-oriented SDT applications such as infection control and pandemic forecasting, the term *societal digital twin* highlights a broader vision. It refers to a digital twin framework designed to promote not only reactive measures during outbreaks, but also proactive, society-wide health services—such as immunization planning, long-term public wellness modeling, and infrastructure resource optimization. Thus, SDT covers the full spectrum from emergency healthcare to sustained societal well-being.

**Fig. 2:**
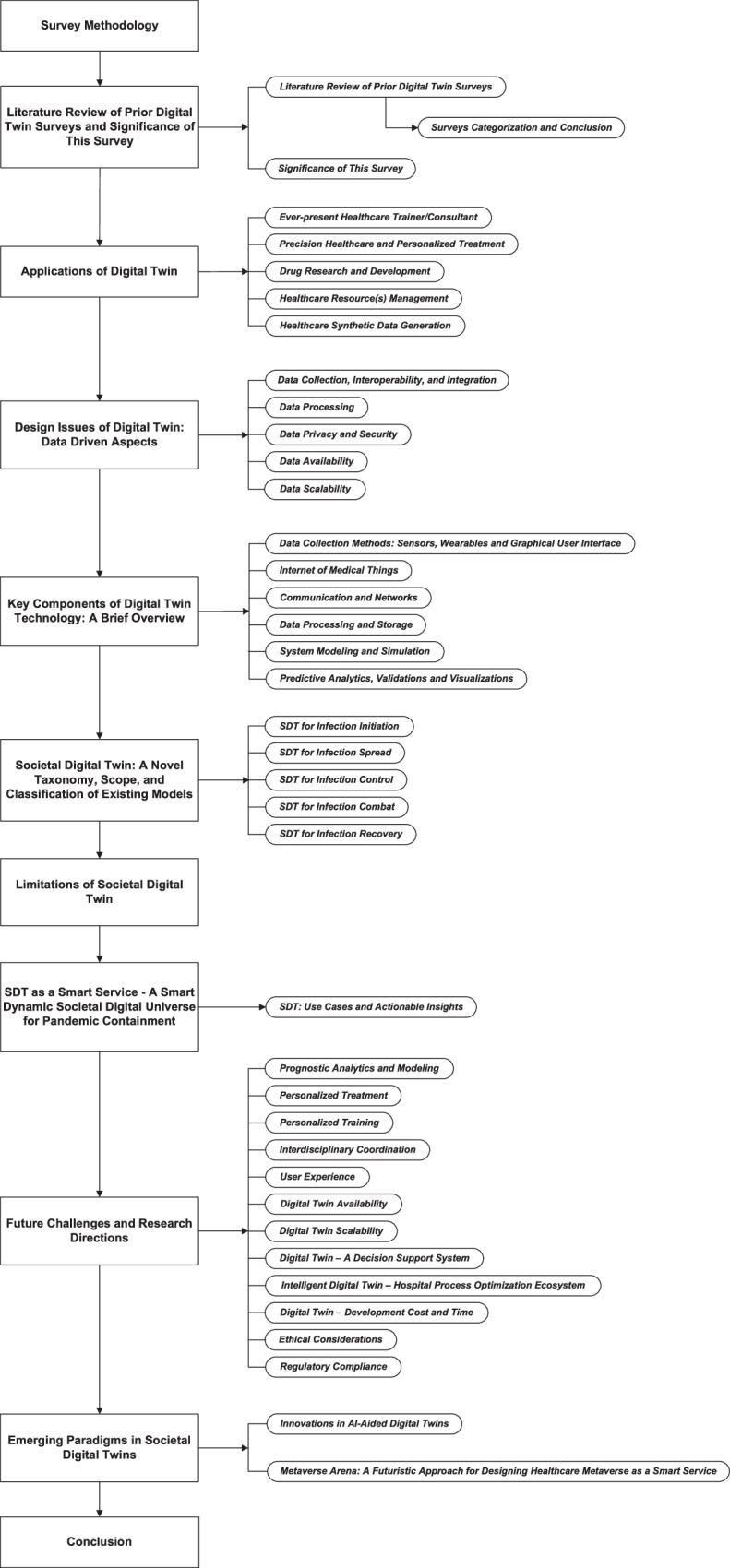
Organization of the manuscript.

Some of these techniques to restrain the COVID-19 pandemic include a city DT^20^, SARS-CoV-2 spread forecast^21^, social distancing^22^, population vaccination^23^, human immune system modeling^24,25^, and so on. The development of such techniques in the recent past exhibits a growing interest within the research community in SDTs. However, to the best of our knowledge, there exists a significant gap in comprehensively categorizing and analyzing the available SDT approaches. To bridge this gap, this survey takes a pioneering step in exploring the SDT landscape for infection management by introducing a novel taxonomy—Rehan’s Taxonomy. The acronyms used in this survey are listed in Table 1, and the organization and contents of this manuscript are depicted in Fig. 2.

**Table 1:** Listing of acronyms with description

Acronym	Description	Acronym	Description
AI	Artificial Intelligence	ABM	Agent Based Model
AR	Augmented Reality	CA	Cellular Automaton/Automata
CDSDT	Clinical Decision Support Digital Twin	CDT	Cloud Digital Twin
CPDT	Comprehensive Personalized Digital Twin	CPS	Cyber Physical Systems
CT scans	Computed Tomography scans	DBMS	Database Management Systems
DT	Digital Twin	DTaaS	Digital Twin as a Service
EDT	Edge Digital Twin	EHR	Electronic Health Records
EP	Electrophysiology	FDT	Fog Digital Twin
FHIR protocol	Fast Healthcare Interoperability Resources protocol	FL	Federated Learning
GSDF	General Survey Design Framework	GUI	Graphical User Interface
HTTPS	Hypertext Transfer Protocol Secure	HMaaSS	Healthcare Metaverse as a Smart Service
ICTs	Information and Communication Technologies	IDT	Intelligent Digital Twin
IoMT	Internet of Medical Things	IoT	Internet of Things
ML	Machine Learning	MR	Mixed Reality
MRI	Magnetic Resonance Imaging	NPIs	Non-Pharmaceutical Interventions
PDT	Personalized Digital Twin	PRISMA	Preferred Reporting Items for Systematic Reviews and Meta-Analyses
R&D	Research and Development	RMDT	Resource Management Digital Twin
SDL	Specification and Description Language	SDLPS	Specification and Description Language Parallel Simulator
SDT	Societal Digital Twin	SDTaaSS	Societal Digital Twin as a Smart Service
SDTICom	SDT for Infection Combat	SDTICon	SDT for Infection Control
SDTII	SDT for Infection Initiation	SDTIR	SDT for Infection Recovery
SDTIS	SDT for Infection Spread	SHM	Societal Healthcare Metaverse
SQL	Structured Query Language	SEIR(D) model	Susceptible Exposed Infected Recovered (Deceased) model
TCN	Time Convolutional Network	VR	Virtual Reality

This effort is timely and aligns with the increasing global focus on scalable, ethical, and intelligent digital health preparedness. The key contributions of this paper are summarized as follows:

- A structured review of existing SDT-related literature, organized using an application-oriented methodology informed by PRISMA principles, helping to identify gaps and trends across digital health domains.
- The introduction of new service-oriented concepts that enable dynamic and accessible deployment of SDT ecosystems through smart, on-demand models.
- A forward-looking classification model (Rehan’s Taxonomy) that contextualizes SDT applications along the infection response continuum.
- Practical insights into data-driven design challenges, enabling technologies, and application-specific considerations critical to the real-world implementation of healthcare-oriented Digital Twins.
- A synthesis of emerging paradigms—such as AI-aided, federated, and human-centric DTs—and a vision for future research, including the proposed Healthcare Metaverse as a Smart Service.

## Survey methodology

This study adopts a structured survey methodology, inspired by systematic review practices—particularly the Preferred Reporting Items for Systematic Reviews and Meta-Analyses (PRISMA) framework^26^. While PRISMA is primarily intended for medical and clinical meta-analyses, we apply its core principles—transparency, reproducibility, and systematic filtering—to guide our review of literature related to SDTs.

### Scope and objective

Our aim is to systematically identify, classify, and analyze academic and gray literature at the intersection of Digital Twins, Artificial Intelligence, public health preparedness, and emergency response systems.

### Databases and sources

We searched across multiple scientific databases, including IEEE Xplore, ACM Digital Library, Scopus, PubMed, and Google Scholar. Searches were conducted between January 2024 and May 2025, including both recent and foundational literature.

### Search strategy

We used combinations of relevant keywords such as “Digital Twin”, “Societal Digital Twin”, “Health Digital Twin”, “Generative Digital Twin”, “AI-aided DT”, “public health”, “epidemic”, “pandemic modeling”, and “resilience planning”. Boolean connectors (AND, OR) were used to combine terms.

### Inclusion and exclusion criteria

We included papers that (i) focused on population-scale or public-oriented DTs, (ii) applied or discussed AI techniques in DT contexts, and (iii) provided technical or conceptual insights relevant to emergency response, health modeling, or infrastructure resilience. Articles not available in English, lacking full-text access, or unrelated to DT or public health were excluded.

### Filtering and classification

After initial identification, duplicate entries were removed. Titles and abstracts were screened, followed by full-text reviews. A total of 70 studies were shortlisted and organized thematically using the proposed Rehan’s Taxonomy, which was iteratively refined during synthesis.

### PRISMA alignment

**Fig. 3:**
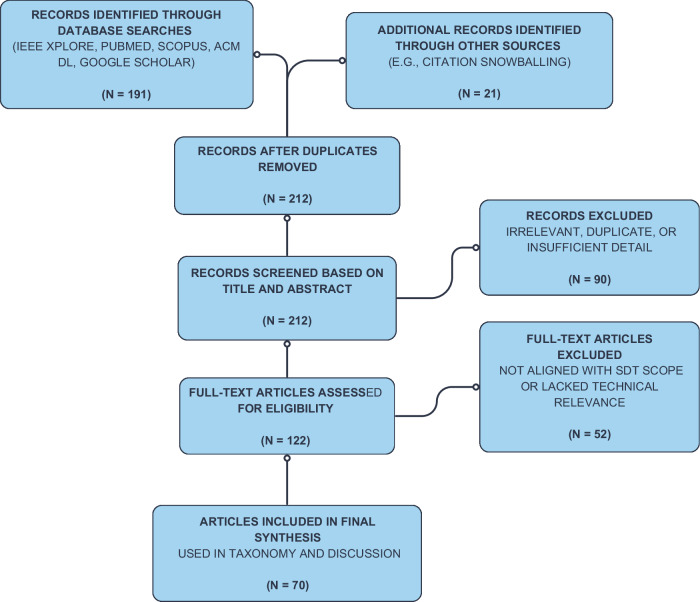
PRISMA-style literature filtering and selection process.

Although not a clinical review, our process aligns with PRISMA principles in terms of clearly defined eligibility criteria, transparent search protocols, and structured synthesis of findings. A simplified PRISMA-style diagram summarizing the selection process is provided in Fig. 3.

## Literature review of prior digital twin surveys and significance of this survey

The primary focus of this section is to exhaustively and conclusively review previous healthcare-related DT surveys, and subsequently highlight the significance of this survey. The motivation is primarily influenced by the *General Survey Design Framework (GSDF)*^27^.

### Literature review of prior digital twin surveys

The authors in ref. ^7^ discuss the role of DT technology in optimizing activities across various healthcare sectors, such as early disease diagnosis, personalized treatment planning, optimizing hospital processes, advancing the pharmaceutical industry, and enabling digital monitoring using wearable technologies. Furthermore, the survey discusses relevant projects in each healthcare domain and briefly summarizes the results. Finally, it presents future insights emphasizing the role of DT technology in surgical planning, physiotherapy treatment, precision medicine, organ transplantation, and resource management in hospitals.

In^28^, the authors discuss the prospects of DT technology in medicine. They highlight numerous implementations of DT technology across various medical fields including orthopedics, cardiovascular disease, and pharmacy. The article underscores the capability of DT technology for precise diagnosis, risk prediction, personalized treatment, and real-time monitoring of patients. It emphasizes numerous challenges associated with DT technology regarding data collection and fusion, simulation accuracy, and socio-ethical concerns. The article proposes that advancements in Big Data, IoT, and Artificial Intelligence (AI) may further exploit the technological advantages of DT technology. In the future, a thorough DT framework for the human body could facilitate early disease prediction for humans, especially for elderly people.

The authors in ref. ^29^ discuss the conceptualization and utilization of DT technology in various areas of healthcare, such as personalized treatment, clinical trial design, and hospital process optimization. They outline a brief conceptualization, working mechanism, data generation methodologies, and technologies for realizing DT technology in healthcare. Furthermore, numerous ethical and technical concerns regarding data bias, data privacy and security, data gathering, data handling, and user interface design are discussed. Finally, some social challenges regarding DT accessibility, equal representation, and healthcare staff downsizing are presented.

In^30^, the authors discuss the potential of DT in administering healthcare systems. They outline key findings regarding DT technology from numerous healthcare domains, such as safety oversight, operational supervision, data governance, healthcare administration, and individual welfare enhancement. The article argues that DT technology may provide a viable solution for real-time monitoring, distant user testing, and devising patient-centric care approaches. However, it may suffer from numerous challenges, such as data privacy, security, sharing, and ownership. Finally, the review emphasizes the necessity of interdisciplinary research to explore the viability of, and address obstacles in, implementing DT technology in the medical sector.

The authors in ref. ^31^ introduce a DT model for providing healthcare assistance across various stages of life such as preconception care, lifetime healthcare, and the afterlife stage. They give a historical overview and clarify misinterpretations about DT technology. The survey proposes Digital Twining everything as a healthcare service model enabling Industry 4.0. It discusses the role of DT in carrying out equality, resource management, and research in healthcare. The survey outlines future challenges associated with DT technology, such as digital disparity, equitable availability, privacy, security, interoperability issues, and disparate standards. Finally, the necessity of uniform standards and legislation for the resilient advancement of DT technology in the medical domain is emphasized.

In^13^, the authors discuss numerous DT applications in personalized treatment. The survey aims to explore the methods and data sources for building DT systems for numerous medical sectors. To understand foundational methodologies, several case studies pertaining to the artificial pancreas, cardiology, single-cell flux analysis, protein-DNA interplay, oncological clinical findings, medication efficacy, and therapeutic repositioning for COVID-19 are presented. The survey outlines the challenges of DT applications and emphasizes solutions for issues such as data collection, computation, integration, and patient data privacy to achieve digital transformation.

The authors in ref. ^32^ discuss numerous applications and challenges of DT technology in medicine. They recommend the role of DT models’ synergy in optimizing clinical processes and integrating new technologies. The survey highlights numerous clinical applications of DT technology involving cardiovascular disease, surgery, pharmacy, orthopedics, and COVID-19 management. The survey suggests the potential of DT technology in dynamic monitoring, precision prognosis, personalized treatment, and forecasting health-disease states. It argues that advancements in IoT, Big Data, and AI have enhanced DT applications in medicine. However, numerous technical and ethical concerns still require attention.

In^33^, the authors discuss wide-ranging applications of DT technology in various healthcare domains, including personalized treatment, pandemic handling, bio/pharmaceutical manufacturing, and resource management. They describe how DT carries out personalized treatment by developing patient-oriented models for early disease forecasts and treatment scheduling. Pandemic handling requires pandemic initiation and spread prediction for planning effective response mechanisms proactively. DT optimizes the process for bio and pharmaceutical manufacturing by developing biologically engineered products and drugs, respectively. Healthcare resource management involves the efficient assignment and operation of hospital/clinical resources. Besides that, the survey discusses Machine Learning (ML), security, and ethical issues of DT technology in the medical domain. Finally, it outlines future challenges, namely efficient computing, privacy, integration, regulations, and stakeholder involvement.

The authors in ref. ^34^ explore the potential of DT technology in revolutionizing healthcare in the metaverse era. They discuss DT in personalized and precision medicines. The survey examines cutting-edge approaches and datasets, along with various online platforms for creating DTs across different domains. The review highlights numerous future research challenges regarding data privacy, security, interoperability, scalability, validation, and ethical/regulatory concerns. By overcoming these challenges, the real benefits of DT technology in the healthcare sector may be ensured.

In^35^, the authors explore applications of DT technology in improving personalized treatment, predictive analytics, clinical operations, and facilitating training and simulation. DT ensures individualized care by prescribing treatments based on comprehensive patient profiles. It may lead to effective personalized treatment strategies that achieve promising results. Through predictive analytics using real-time and historical data of patients, a DT may achieve early disease forecasting. It may enable healthcare professionals to adopt preventive measures proactively to address health-related issues. DT technology may optimize clinical operations by streamlining hospital processes, resource demand patterns, and identifying limitations in managing healthcare resources effectively. By facilitating training and simulation, the skills and decision-making abilities of healthcare professionals may be improved, leading to better treatment for patients.

#### Surveys categorization and conclusion

The aim of this section is to highlight the use case domains of previously discussed health-related surveys under three main categories, i.e.:

1. *Comprehensive Personalized Digital Twin (CPDT):* A CPDT embodies the capabilities of Personalized Digital Twin (PDT) and Clinical Decision Support Digital Twin (CDSDT). It employs prognostic analytics and medical expertise to make sophisticated decisions about patient well-being. Like PDT, it performs immediate decision-making for day-to-day personalized treatment, e.g., predicting insulin dosage adjustments for a patient based on glucose levels. Following CDSDT, it supports healthcare professionals in making informed decisions for long-term patient well-being, such as prescribing medical dosage adjustments. Likewise, it streamlines personalized health-related resources for managing patient treatment during routine and emergency situations, such as planning surgical procedures.
2. *Resource Management Digital Twin (RMDT):* An RMDT aims to optimize healthcare operational resources or streamline workflow processes for the benefit of the community. For example, optimized resource management in hospitals may be achieved by effective forecasting of patient influx management, staff scheduling, and parking allocation during routine and emergency situations.
3. *Societal Digital Twin:* Given the global devastation of SARS-CoV-2 (COVID-19), the concept of a comprehensive SDT framework is initially conceptualized in this paper with the core purpose of deploying DT technology to elevate societal well-being on a broader scale, such as analyzing social interactions to predict the outbreak of infections at local or global levels and suggesting measures for controlling and combating infections, e.g., promoting vaccination campaigns or developing response systems for managing infectious disease outbreaks.

Considering the scope of the above-mentioned application areas, such as CPDT, RMDT, and SDT, the survey literature is organized in Table 2. A critical analysis reveals that the majority of existing surveys focus on DT applications belonging to the CPDT and RMDT categories. However, to the best of our knowledge, only a few surveys^13,30,33^ address limited DT applications targeting infectious diseases, providing only partial coverage of this critical area. This survey is unique compared to the above-mentioned surveys, as it exclusively focuses on Societal Digital Twin (SDT) approaches and comprehensively reviews them under a novel taxonomy. Given the profound socio-economic and political impacts of COVID-19, both during and after the pandemic, there is a pressing need for a thorough review in this area of research. To bridge this gap, this survey aims to provide a solid foundation and inspire the research community to explore SDT approaches to promote healthcare innovation using DT technology and proactively address future healthcare challenges, such as those posed by COVID-19.

**Table 2:** Digital twins in the various areas of digital healthcare

Survey	Year	DT area(s) of interest	CPDT	RMDT	SDT
T. Erol et al.^7^	2020	Digital patients, pharmaceutical industry, hospital processes, and wearable technologies	*✓*	*✓*	
T. Sun et al.^28^	2022	Precise diagnosis, real-time monitoring, and personalized treatment	*✓*	*✓*	
P. Armeni et al.^29^	2022	Personalized medicine, clinical experimentation, and hospital operations management	*✓*	*✓*	
S. Elkefi et al.^30^	2022	Safety oversight, operational supervision, data governance, healthcare administration, and individual welfare enhancement	*✓*	*✓*	*✓*
H. Hassani et al.^31^	2022	Digital patients, precision medicine, hospital processes, and wearable technologies	*✓*	*✓*	
C. Meijer et al.^13^	2023	Personalized/precision medicine	*✓*		*✓*
T. Sun et al.^32^	2023	Medicine, patient dynamic monitoring, and precision healthcare	*✓*	*✓*	
S. Ghatti et al.^33^	2023	Precision healthcare, pandemic response, pharmaceutical industry, and machine learning/modeling	*✓*	*✓*	*✓*
M. Turab et al.^34^	2023	Personalized and precision medicine	*✓*	*✓*	
A. Vallée et al.^35^	2023	Personalized treatment, clinical operations, and healthcare professionals’ training	*✓*	*✓*	

*CPDT* Comprehensive Personalized Digital Twin, *RMDT* Resource Management Digital Twin, *SDT* Societal Digital Twin.

### Significance of this survey

This survey provides a comprehensive review of *digital twin* technology in healthcare, with a primary emphasis on *societal digital twins*. The key insights of this review are summarized as follows:

- *Systematic Literature Organization:* This survey systematically organizes and evaluates recent healthcare-related digital twin survey literature using an application-oriented methodology. It categorizes existing survey literature into three classes, as presented in Table 2. This structured classification highlights the scarcity of existing surveys addressing SDT-related approaches and underscores the need for a comprehensive review in this emerging area of healthcare innovation.
- *Novel Taxonomy of SDTs:* To the best of our knowledge, this is the first comprehensive review to examine a significant number of SDT approaches under a novel taxonomy, classifying SDTs into five categories, namely infection initiation, spread, control, combat, and recovery. To enhance clarity, the scope of each category is defined, and relevant approaches are briefly analyzed in terms of functionality, validation strategies, generalizability, and limitations, thereby identifying key challenges.
- *SDT as a Smart Service (SDTaaSS):* This survey introduces the concept of a *smart, dynamic societal digital universe* for pandemic containment, termed SDTaaSS. As a forward-looking healthcare innovation, SDTaaSS represents a smart service-oriented paradigm that shifts the complexity of system development to service providers. It empowers non-technical stakeholders to deploy SDTs on demand via a smart subscription-based model.
- *Applications, Data-Driven Design Issues, and Key Components of DT:* The review offers an in-depth exploration of DT applications in the medical domain, highlighting practical implementations in healthcare. It further discusses data-driven design issues and addresses challenges related to effective data utilization in healthcare-related DTs. Additionally, it reviews core DT components and emphasizes the role of enabling technologies in developing functional healthcare DT systems.
- *Future Research Directions and Emerging Paradigms:* The survey presents a detailed discussion of open challenges and future research directions in healthcare-related DTs. It highlights key issues such as interdisciplinary coordination, ethical considerations, and regulatory compliance. Moreover, it explores emerging innovations in *AI-aided digital twins* and proposes the concept of the Healthcare Metaverse as a Smart Service (HMaaSS).

## Applications of digital twin

**Fig. 4:**
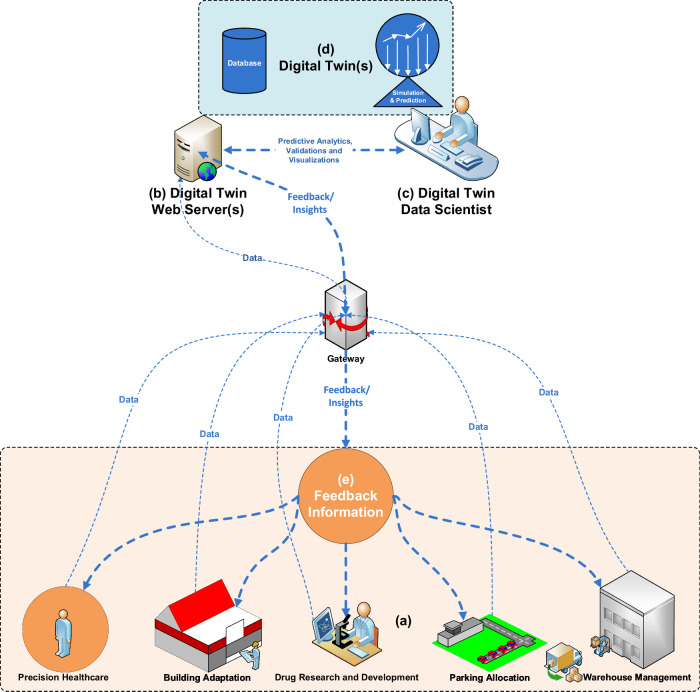
Digital twin applications in healthcare. This figure illustrates the data flow in various application areas within the healthcare domain. **a** Represents numerous physical world systems such as precision healthcare, building adaptation, drug research and development, parking allocation, and warehouse management sending digital data for predictive analytics and visualizations. **b** Digital Twin Web Server(s): Depicts a gateway for routing information to the central Digital Twin web server(s) for storing and processing the digital information for prediction and simulation. **c** Digital Twin Data Scientist: Represents a Digital Twin data specialist who uses AI and ML approaches to visualize the future behaviors of real-world entities, understand healthcare challenges, fine-tune the Digital Twin model for improved predictive analytics, and validate these future behaviors of real-world healthcare systems. **d** Digital Twin(s): Shows the Digital Twin of the physical world systems. **e** Feedback Information: Displays response metrics containing real-time insights to better equip the physical-world systems to tackle future healthcare challenges.

DT technology exhibits a wide range of applications across nearly every area of the medical industry, ranging from personalized healthcare and clinical digital support to hospital resource management and societal well-being. This section discusses the significant applications of healthcare-related DT technology, as depicted in Fig. 4 and briefly described below:

### Ever-present healthcare trainer/consultant

DT may serve as an ever-present healthcare trainer, supporting both the maintenance of a healthy lifestyle and the rehabilitation or recovery of patients. Based on the past history and current habits of users, it may recommend suitable diet plans, daily exercise routines, and leisure activities accordingly. Being readily available, users may even discuss minor healthcare concerns with their personalized DT and receive immediate feedback or suggestions within the given context. Consequently, users may feel more cared for, valued, and secure, ultimately improving their overall experience and well-being. In the case of a minor issue, a personalized DT may instantly suggest changes to diet or exercise plans, whereas in more critical situations, it may automatically notify a physician about potential risks to proactively enable preventive interventions.

### Precision healthcare and personalized treatment

Precision healthcare focuses on prescribing *precision medicine* for personalized well-being rather than recommending a one-size-fits-all treatment, as carried out in traditional *standardized medical treatment*. More specifically, precision medicine aims to prescribe the appropriate treatment to each individual at the optimal time^9^. By embodying genetic makeup, historical health records, living habits, and current health conditions of an individual, DT may identify numerous biomarkers for better understanding and diagnosing the cause of a disease. Such a decision support system may help a physician prescribe *precision medicine* to the patient.

Predictive modeling has an important role in personalized preventive interventions. Using predictive modeling in the DT environment, disease advancement patterns may be identified preemptively, and preventive measures may be applied proactively^35^. By doing so, physicians can customize treatment strategies to restrain or halt disease advancement, thereby minimizing health risks for patients. Another example of personalized treatment is the cloud healthcare system, CloudDTH^36^, which is designed to examine, diagnose, and estimate health metrics for elderly individuals.

By predicting and evaluating potential outcomes, healthcare risks may be avoided, and healthcare quality can be improved^33^. In critical situations, DT can initially be used to test drug delivery or surgical treatments^7,33^. Another compelling application of DT is in organ donation, whereby suitable organ recipients may be matched through DT-based testing, matching, and simulations^31^. Such advancements may significantly enhance the success rate of organ donation, thereby contributing to saving countless lives.

### Drug research and development

Drug Research and Development (R&D) is often a complex task. It involves numerous clinical trials that are often slow-moving and costly, partly because enrolling participants requires both the availability of suitable target groups and their willingness to participate^29^. Thanks to the DT paradigm for running tests, analyzing data, and verifying test theories^35^, thus providing robust solutions to speed up drug R&D. For instance, the target population’s DT may be consulted for enrollment in the clinical trials of drug exploration. Being readily available 24/7 and easily replicable, selected DTs may accelerate the drug exploration process.

Furthermore, DT technology may enable the use of *Digital Information from* the deceased to understand the exact cause(s) of death and facilitate further research in drug development to address those causes. Doing so may also assist in enhancing the drug-related knowledge base, deciphering complex, refractory, and intractable diseases, and developing innovative medications to facilitate personalized treatment in the future.

### Healthcare resource(s) management

Under normal conditions, healthcare-related resources (e.g. staff, beds, equipment, pathways, and parking) are managed using pre-allocated schedules, which systematize when and how these resources are employed. This may make complex processes and procedures more straightforward and orderly in both hospitals and clinics. However, during emergency situations, a surge in patients often requires reallocating healthcare-related resources and creating or adjusting schedules more quickly, often in real time. Therefore, healthcare-related resource allocation may become tenuous, compromising, and challenging. To handle such complexities, DT may provide a reasonable solution for hospital resource management^7^. It may assist in healthcare professionals training^35^, emergency resource scheduling^37^, pathways prediction^38^, facility management^39^, building operations^40,41^, and so on.

Outlined below are the significant applications of DT in medical resource management:

- *Patients Management:* DT may assist in intra-hospital patient management such as organizing patient flow^30,31^. However, during a crisis situation, such as pandemic, managing patients in hospitals becomes highly challenging. A viable solution to address such a situation may be provided by a City-RMDT. A City-RMDT may embody RMDTs of numerous hospitals in a city and would be intelligent enough to predict patient influx at a given time across different hospitals in a city. By considering various factors such as pandemic situation, traffic movement, and historic information, a City-RMDT may provide public guidance beforehand regarding increased waiting times at various hospitals and alternative solutions, such as visiting the best hospital nearby based on user preferences.
- *Employee/Equipment Organization:* A DT may be modeled based on the skill sets of the hospital staff or the operational status and performance of medical equipment^33^. Using AI and ML techniques, DT may predict the best possible staff allocation in hospitals and preemptively revise the staff roster. Likewise, through predictive maintenance, DT may forecast potential issues in medical equipment^35^ and suggest replacing devices to achieve optimal performance in hospitals and clinics.
- *Personnel Training:* The seamless availability of DT technology may provide a valuable resource for equalizing access to medical education^31^ and producing skilled healthcare professionals, regardless of geographical location. Providing hands-on, state-of-the-art training in DT environment may enhance the confidence and expertise of healthcare professionals, preparing them to contribute to patient safety^35^ and the proper functioning of hospitals and clinics. Being a one-time investment, the role of DT in leveraging repetitive training for healthcare workforces may have far-reaching implications in saving human resource training budgets. During the pandemic scenario, DT may offer robust solutions for training paramedic teams on an emergency basis. By doing so, any shortage of healthcare professionals may be efficiently and urgently met, thereby strengthening and optimizing the healthcare processes.
- *Building Adaptation:* In the modern era, it is crucial to construct hospitals in a manner such that the building structure can be easily adapted to accommodate the growing demand for patient care in emergency situations. A DT may provide a reasonable solution in this regard and may assist in creating a flexible building information model of a hospital. Consequently, it may accommodate the rising demand for patient care in a hospital through small localized architectural adjustments within the hospital premises.
- *Warehouse Management:* Effective healthcare services require a continuous flow of medical supplies, such as gloves, bandages, syringes, surgical instruments, drugs, and diagnostic machines. Proper procurement and management of medical supplies in hospital warehouses are crucial for managing supply shortages that were readily observed during global pandemics like COVID-19. By enabling real-time monitoring and coordination between hospitals and medical suppliers, DTs may predict and mitigate imbalances between supply and demand, thus ensuring efficient healthcare delivery to society at large.
- *Parking Allocation:* Each hospital has a limited parking space to accommodate visitors. However, during emergencies or pandemic situations, patient and guest influx increases tremendously in hospitals. It may cause a rush and traffic congestion on hospital premises. Such a situation may critically impact the associated management processes in hospitals. A DT may preemptively predict the occurrence of any such congestion situations in the vicinity of a hospital. As a result, the visitors may be guided beforehand about alternate solutions for streamlining the hospital operations.

### Healthcare synthetic data generation

Properly modeling the physical environment may enable DT technology to deeply understand the complexities of real-world systems and effectively predict solutions for unknown use cases. Considering the potential of DT technology, it may serve as a promising resource in healthcare-related synthetic data generation. Such valuable artificial data may serve as a viable source for training ML models on prospective scenarios experiencing data unavailability or insufficiency. For example, in a cardiovascular DT^42^, synthetic PhotoPlethysmoGram (PPG) data is generated using varying blood flow and pressure measurements that may replicate the cardiovascular system and help in training machine learning models.

## Design issues of digital twin: data driven aspects

The digital twin is a data-oriented methodology where data plays a crucial role in modeling and simulating real-world objects or systems. Therefore, it is paramount to navigate through the main data-driven design issues, such as data collection, interoperability, integration, processing, computing, privacy, security, availability, and scalability. A brief overview of data-driven challenges in healthcare-related DT is outlined below:

### Data collection, interoperability, and integration

Digital data in healthcare exists in numerous forms, including Electronic Health Records (EHR), data from wearable and IoT devices, patient reports, Magnetic Resonance Imaging (MRI), cardiac Electrophysiology (EP), and Computed Tomography (CT) scans. Data collection and integration from such heterogeneous data sources are critical obstacles^13^. This is because each data type has distinct challenges in terms of integration, interpretation, and standardization^13^, thereby making operations on diverse data an arduous task^29^. Therefore, creating a coherent data source (from multiple sources) to provide a uniform data view within a DT environment is a daunting issue. To accomplish this, it is crucial to establish common data standards that may facilitate seamless communication and achieve interoperability, integration, and compatibility among various data sources and DT environments. A prominent standard for electronically sharing medical information is the Fast Healthcare Interoperability Resources (FHIR)^43^ framework, specified by HL7^44^. The FHIR standard enables data interoperability between DT and medical systems by providing an API to retrieve data from EHRs in a standardized format.

### Data processing

**Fig. 5:**
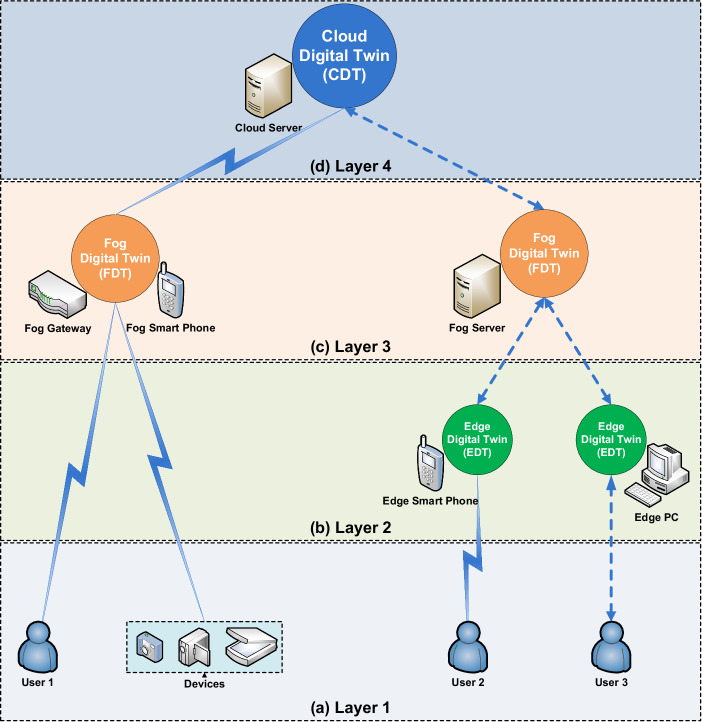
Data processing-oriented methodologies in healthcare digital twin. This figure depicts hierarchical data processing and storage methodologies based on healthcare application requirements and resource availability. **a** Layer 1: Users and medical devices send data to Edge, Fog, and Cloud-based network architectures. **b** Layer 2: The EDT operates in close vicinity to data sources, providing real-time data analytics with minimal processing and low communication delays. Local processing allows EDT to conserve bandwidth and offer the highest data security and privacy against threats. **c** Layer 3: The FDT resides at the intermediate network level and involves the collaborative analysis of data from various EDTs. It also performs data preprocessing, filtering, and compression before sending refined information to the network core, conserving network bandwidth. FDT requires more processing power, introduces communication delays and necessitates higher security/privacy measures than EDT. **d** Layer 4: The CDT is located at the network core, providing centralized data processing and storage. While it offers the fastest data processing, it introduces higher communication delays and consumes the most network bandwidth. Furthermore, stronger security and confidentiality measures are needed to safeguard information sent to the CDT.

DT holds significant promise as a future healthcare resource, facilitating seamless data processing and enabling the forecasting of medical conditions for users. However, when serving solely as a centralized resource, a DT framework may exhaust network resources in terms of network bandwidth, data processing, and storage. It may induce network delays, privacy and security concerns, and challenges in real-time data analysis. Hence, it is essential to employ a hierarchical methodology for data storage and processing within the DT architecture, tailored to the unique nature of healthcare applications and the availability of network resources. A promising solution is to classify healthcare-related DT systems into edge-, fog-, and cloud-based network architectures, as depicted in Fig. 5 and summarized below:

- *Edge Digital Twin (EDT):* An EDT operates in close proximity (Edge) to the data source, ensuring low latency for network communication, real-time predictive analytics, high security/privacy, and network bandwidth conservation. For example, data on a patient’s daily water intake can be processed and stored on the EDT on the patient’s mobile phone.
- *Fog Digital Twin (FDT):* An FDT resides within the intermediate network for decentralized data processing. With more computing power than EDT, FDT can process and analyze data from numerous edge data sources in more complex structures. Additionally, it may preprocess, filter, or compress the data (e.g., medical images transmitted to the network core) to conserve network bandwidth. An FDT provides greater computing power, faster data processing, and enhanced prognostic analytics than an EDT, while still preserving network bandwidth. For instance, data from sensors, IoT devices, or wearables can be analyzed on the FDT via the patient’s mobile phone or a home area network gateway. However, residing in the intermediate network, FDT may experience higher communication delays and increased bandwidth consumption compared to EDT.
- *Cloud Digital Twin (CDT):* A CDT is centrally located at the core of the network and remotely performs health-related data storage, management, and processing activities. It embodies fast data computing power and may conduct data analytics on the overall network data. However, it may experience more communication delays and consume more network bandwidth than both FDT and EDT. Additionally, sending data to a CDT requires proper implementation of encryption and security measures. A CDT may provide insights into population growth rates as well as the proliferation of communicable diseases or epidemics within the community.

### Data privacy and security

To avoid information exploitation and facilitate information exchange^34^, a DT must ensure high standards of privacy and security for handling sensitive healthcare information. It is paramount to establish data protection measures at the organizational level^33^ and to keep users well-informed about the intrinsic privacy and security measures for enhancing their trust in the DT system. Achieving this requires sophisticated encryption algorithms^34^ (to safeguard data during transfer and storage), robust access control mechanisms^34^ (to restrict unauthorized retrieval of sensitive information), anonymization techniques (to protect patient identities), regular software updates (to mitigate evolving threats), and routine security audits (to identify vulnerabilities in the DT systems). Additionally, various techniques, such as Hypertext Transfer Protocol Secure (HTTPS), blockchain-based Hyperledger Fabric technology^45^, trusted computing environments like Intel’s SGX^46^, Homomorphic Encryption^47^, advanced AI, and Federated Learning (FL) techniques^33^, may be used to enhance data security and privacy.

### Data scalability

The enormous healthcare benefits of DT technology will soon make it an integral part of futuristic healthcare. The growing adoption of healthcare DT technology will simultaneously induce a high volume of data generation, requiring faster data exchange between the physical and digital worlds. This may render the prevailing resources scarce to accommodate the technology shift, potentially inducing network performance bottlenecks in terms of bandwidth, storage, and processing. As a result, employing traditional DT systems at the network core or in the cloud may result in slowdowns or halts. It may induce delayed responses to healthcare applications and undermine the core DT value of providing real-time predictive analytics to healthcare systems.

Data scalability and resource scarcity issues in the DT environment may be addressed by decentralized data processing (edge/fog computing), intelligent compression (data deduplication), data archiving, advanced AI analytics, and FL approaches. Implementing these strategies may provide bandwidth conservation, effective data storage and management, intelligent resource allocation, and parallel computing, thereby enabling real-time analytics and forecasting in next-generation DT systems. Therefore, there is a need to devise new approaches for resource optimization and workload distribution to handle inherent issues and achieve optimized communication in next-generation healthcare DT systems.

## Key components of digital twin technology: a brief overview

**Fig. 6:**
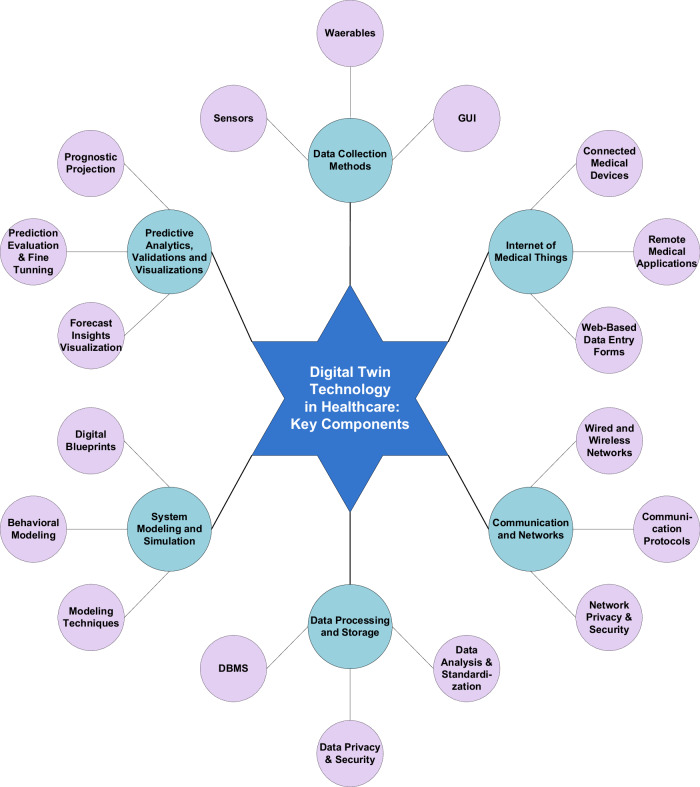
Key components of digital twin technology in healthcare. This figure shows the key components of DT technology in healthcare. Data Collection Methods involve gathering digital biomarkers or procedural data using sensors, wearables and GUIs. The Internet of Medical Things creates an online network of medical devices, enabling real-time and remote healthcare. Communication and Networks describe various data communication protocols and technologies that facilitate robust, safe and secure healthcare services within the SDT ecosystem. Data Processing and Storage concentrate on converting diverse datasets into a standardized form for comprehensive data analysis and storage. System Modeling and Simulation focus on generating digital blueprints of real-world systems, replicating their intrinsic behaviors and responses. Predictive Analytics, Validations and Visualizations entail forecasting and visualizing future behaviors of real-world systems to effectively handle healthcare challenges.

Modeling and simulating a digital duplicate of a physical-world entity is a complex and arduous task. It involves extensive system development intricacies and requires hands-on system implementation skills. Discussing complex system-level engineering of a healthcare-related DT is beyond the scope of this work. However, it is essential to summarize the basic components of a healthcare-related DT as depicted in Fig. 6. Some of these components are discussed below:

### Data collection methods: sensors, wearables and graphical user interface (GUI)

Sensors are either embedded in the medical devices or integrated into wearables (such as smartwatches), facilitating the tracking of essential wellness indicators like heart rate, blood pressure, and body temperature, both in the clinical settings and during routine life. They can seamlessly collect digital biomarkers, including sleep patterns, breathing patterns, voice patterns, movement patterns, and so on, representing real-time information about a patient’s physiological and behavioral condition. Such data is critical in empowering patients to maintain a healthy lifestyle. It also aids physicians in understanding disease progression, implementing preventive measures more effectively, and customizing treatment plans.

However, many times, automated data collection needs to be complemented with manually entered procedural data, within both clinical and individual patient settings. To achieve this, well-designed and interactive Graphical User Interfaces (GUIs) provide a viable solution. On the one hand, GUIs empower patients in their health journey by enabling them to input essential health details, monitor, and analyze their personalized health records. On the other hand, they assist physicians in visualizing, analyzing, and making informed decisions about preventive measures and personalized treatments.

### Internet of medical things (IoMT)

IoMT corresponds to the network of medical devices connected to the internet. These devices are equipped with sensing and processing capabilities and are capable of communicating with each other over the internet. IoMT devices serve various purposes, including patient health monitoring (such as tracking blood glucose levels or measuring blood pressure) and delivering treatment in remote or underdeveloped areas. Additionally, a web form that allows the entry and transmission of healthcare-related data over the internet may be considered a loosely coupled form of IoMT.

### Communication and networks

Numerous communication protocols may be employed to facilitate efficient communication in healthcare-related DT networks. Some of the commonly used protocols may include Message Queuing Telemetry Transport (MQTT) for real-time healthcare data exchange^45^, Constrained Application Protocol (CoAP) for communication in healthcare IoT networks, and HTTPS for securely transmitting healthcare information over the internet. These communication protocols can operate over both wired and wireless networks, such as Zigbee, Bluetooth, Ethernet, or Wireless-Fidelity (Wi-Fi), depending on the specific application requirements.

Optimizing network performance is crucial for healthcare applications. It requires efficient management of network resources and effective resolution of network challenges related to bandwidth, storage, latency, throughput, and security. To achieve network efficiency, both centralized Cloud DTs at the network core and Edge/Fog DTs on local/intermediate networks may be deployed based on application requirements. This approach can enable load balancing, congestion avoidance, and bandwidth conservation, thus facilitating robust healthcare delivery in next-generation DT networks.

Efficient and reliable communication relies on maintaining high standards of network security and privacy. DT networks can be secured using advanced encryption and access control mechanisms. Meanwhile, network privacy may be ensured through advanced authentication and anonymization techniques. To facilitate interoperability among network devices, protocols like FHIR and Data Distribution Service may provide a reasonable solution.

### Data processing and storage

Digital twins may receive data from diverse sources, such as sensors, patient reports, IoMT, cardiac EP devices, and medical imaging equipment, including MRI and CT scans. Given the substantial variation in the form and structure of *digital data*, effective and inclusive data processing is essential. By employing common data standards, integration methodology, normalization, and cleaning approaches, data from different sources may be converted into a uniform format within a coherent data source. Doing so may enable DT systems to perform comprehensive data analysis and help healthcare professionals make more informed decisions about patients’ health conditions. Various Database Management Systems (DBMS), such as Oracle DBMS, Microsoft Structured Query Language (SQL) Server, PostgreSQL, MySQL, Google Cloud Storage, MongoDB, Amazon Simple Storage Service (Amazon S3), Neo4j, and InfluxDB, may be utilized for data storage in DTs. Database administrators may be instrumental in implementing privacy and security measures to prevent information leakage.

### System modeling and simulation

A model serves as the computer-generated blueprint of a tangible entity or system, simulating its intrinsic properties, processes, and behaviors. The precision of a model is determined by its ability to imitate the characteristics and functional behaviors of the real-world object. However, properly modeling a real-world entity or system can be a challenging task, requiring sound expertise and a significant time frame to execute. Additionally, real-world objects or systems typically exhibit a variety of independent and dependent behaviors.

Dependent behavior is usually a response to internal or external stimuli, making it more complex to model. Especially when the stimuli are generated by the external system, it may necessitate either parallel modeling of the associated system or artificially modeling stimuli generation mechanisms, which is really a challenging task in the healthcare domain.

Various approaches exist for modeling healthcare-related physical systems in the virtual realm of DTs. These techniques may include Agent-Based Modeling (ABM)^22,48^, discrete event simulation^49^, ML/AI-based modeling^20,24,50^, hybrid modeling approaches^20,21,25,50–52^, and statistical modeling^13,23,24,53^. Given the intrinsic complexities, a DT system’s architect should carefully analyze the modeling requirements of healthcare-related DT systems.

### Predictive analytics, validations and visualizations

In the realm of healthcare-related DTs, predictive analytics plays a pivotal role. It involves a series of steps, such as data collection, cleaning, analysis, and forecasting, to ensure effective information visualizations. Data scientists leverage AI-based statistical techniques and machine/deep learning approaches to forecast future behaviors of the real-world systems for understanding healthcare challenges and suggesting preventive measures accordingly. By continuously validating DT forecasts against the physical-world healthcare system, the DT model can be refined to improve predictive analytics and thereby enhance the medical usage of healthcare systems^34^.

Visualizing the future behavior of DT models is invaluable for proactively providing insights regarding future patterns and trends of healthcare-related data. Numerous techniques are used to visualize healthcare-related forecasts in DT environments, such as graphs, charts, and maps. Commonly used visualization tools include Grafana, Microsoft Excel, and Google Charts.

## Societal digital twin: a novel taxonomy, scope, and classification of existing models

Societal digital twins empower healthcare authorities to implement proactive and preventive strategies for community well-being. These strategies include predicting the spread of infectious diseases, initiating healthcare campaigns, and planning vaccination programs, as well as facilitating preliminary drug development and immune response assessments to combat outbreaks.

**Fig. 7:**
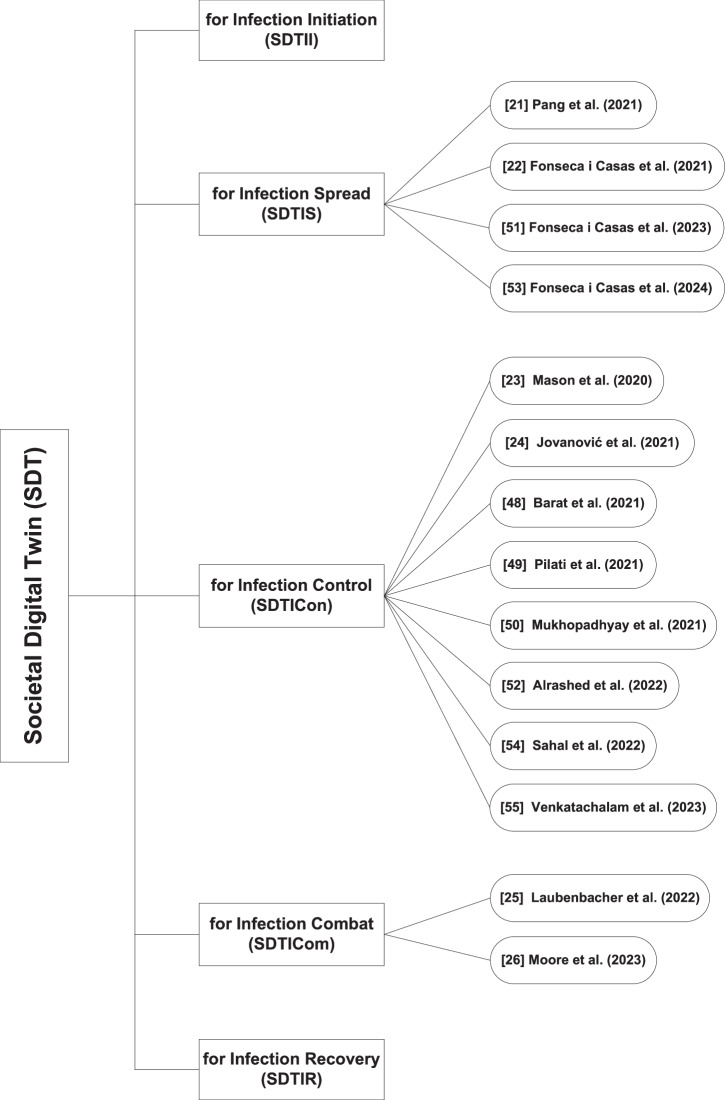
Rehan’s taxonomy and classification of societal digital twin.

Based on operational complexity, SDTs can be classified into five categories, i.e., infection initiation, spread, control, combat, and recovery, as illustrated in Fig. 7. An overview of key characteristics and technological aspects of the SDTs discussed in this section is summarized in Tables 3, 4. The following sections explore the scope and key techniques within each category.

**Table 3:** Article type, data source, and primary insights of societal digital twins

Category	Approaches	Year	Article type			Data source	Primary insights
			Case study	Conceptual framework	Use case		
SDTII	***×***	–	–	–	–	–	–
SDTIS							
	J. Pang et al.^20^	2021			*✓*	COVID-19 tracking project and State Actions dataset	Outlines a collaborative city DT architecture based on Federated Learning for predicting and managing the transmission of COVID-19 pandemic.
	P. Fonseca i Casas et al.^21^	2021	*✓*			Socrata open data source	Predicts and monitors the spread of COVID-19 employing DT methodology.
	P. Fonseca i Casas et al.^51^	2023		*✓*		Catalonia health data on cases, deaths, hospitalizations, and tests	Explores and estimates the influence of non-pharmaceutical interventions on COVID-19.
	P. Fonseca i Casas et al.^53^	2024			*✓*	Wastewater data from SARSAIGUA and detected cases data from ASPCAT	Discusses a joint framework of DT and wastewater analysis for COVID-19 prediction and validation.
SDTICon							
	D. Marti Mason et al.^22^	2020			*✓*		Visualizes the role of physical separation for controlling the propagation of SARS-CoV-2.
	R. Jovanović et al.^23^	2021			*✓*	Daily COVID-19 cases in Belgrade	Explores vaccination as control mechanism for reducing disease prevalence.
	S. Barat et al.^48^	2021	*✓*			Pandemic, census, journals data	Implements ABM simulation for evaluating the effectiveness of region-specific preventive measures on controlling the COVID-19 pandemic.
	F. Pilati et al.^49^	2021	*✓*			Time series data	Development of mass vaccination centers for COVID-19 immunization.
	A. Mukhopadhyay et al.^50^	2021			*✓*	Laboratory oriented real data and VR based synthetic data	Employs deep learning and virtual reality oriented DT for enforcing social distancing measures during the COVID-19 pandemic.
	S. Alrashed et al.^52^	2022		*✓*		COVID-19 spread data in Pakistan	Discusses DT as a resource for reducing the spread of COVID-19.
	R. Sahal et al.^54^	2022		*✓*	*✓*		Employs blockchain DT mechanism for decentralized epidemic alerting.
	I. Venkatachalam et al.^55^	2023	*✓*			Health data from Singapore general hospital	Monitors contact and cluster tracing to infection exposure in the hospital environment.
SDTICom							
	R. Laubenbacher et al.^24^	2022		*✓*		Patient specific data	Presents a prototype of the immune DT for healthcare.
	R. Moore et al.^25^	2023		*✓*		Real-world experimental data	Introduces 1st blueprint of immune system at cellular level as a step towards precision medicine and comprehensive immune system DT for future.
SDTIR	×	–	–	–	–	–	–

× : Unavailability of Approaches.

*SDTII* SDT for Infection Initiation, *SDTIS* SDT for Infection Spread, *SDTICon* SDT for Infection Control, *SDTICom* SDT for Infection Combat, *SDTIR* SDT for Infection Recovery.

**Table 4:** Digital tools, tech solutions, and models of societal digital twins

Category	Approaches	Digital tools				Tech solutions			Data model
		Sensors/IoT	Blockchain	AI/ML	Simulation/ Visualization	Industry 4.0	Metaverse (AR/VR/MR)	CPS	
SDTII	×	–	–	–	–	–	–	–	–
SDTIS									
	J. Pang et al.^20^			*✓*	*✓*	*✓*			TCN and federated learning approaches
	P. Fonseca i Casas et al.^21^			*✓*	*✓*	*✓*			SDL model’s scenario 2.9c
	P. Fonseca i Casas et al.^51^				*✓*	*✓*			Extended SEIRD model i.e. CA-SCVEI_R_I_D_RHUD
	P. Fonseca i Casas et al.^53^				*✓*				Statistical and analytical methods
SDTICon									
	D. Marti Mason et al.^22^				*✓*		*✓*		Agent based model
	R. Jovanović et al.^23^				*✓*				Two-layered statistical model considering the SEIR-V model
	S. Barat et al.^48^			*✓*	*✓*				Agent based model
	F. Pilati et al.^49^	*✓*			*✓*	*✓*			Discrete-event simulation model
	A. Mukhopadhyay et al.^50^			*✓*	*✓*		*✓*		Convolutional Neural Networks (for occupancy estimation) and PyTorch KeypointRCNN (for body posture prediction)
	S. Alrashed et al.^52^								Modified SEIR model
	R. Sahal et al.^54^	*✓*	*✓*	*✓*				*✓*	
	I. Venkatachalam et al.^55^				*✓*				Three-dimensional disease outbreak surveillance system
SDTICom									
	R. Laubenbacher et al.^24^			*✓*		*✓*			Mechanistic models and/or data-driven statistical and ML algorithms
	R. Moore et al.^25^				*✓*	*✓*			Logical modeling approach
SDTIR	×	–	–	–	–	–	–	–	–

× : Unavailability of Approaches.

*SDTII* SDT for Infection Initiation, *SDTIS* SDT for Infection Spread, *SDTICon* SDT for Infection Control, *SDTICom* SDT for Infection Combat, *SDTIR* SDT for Infection Recovery.

### SDT for infection initiation (SDTII)

*Scope:* A SDT for Infection Initiation may predict the onset and origins of infections within society by considering vectors such as mosquitoes, contaminated food or water, and infected individuals.

*Approaches:* In exploring this area of research, a clear scarcity of techniques is observed. Further brainstorming and innovative solutions are required to bridge this research gap.

### SDT for infection spread (SDTIS)

*Scope:* A SDT for Infection Spread may forecast the mechanisms of infection transmission in society, such as overcrowding, travel, and social interactions. It may also anticipate infection proliferation mechanisms, including respiratory droplets, face-to-face contact, and exposure to polluting substances.

*Approaches:* This section explores numerous SDTIS approaches, along with their validation strategies, generalizability, and limitations. Among these, extensions of the Susceptible Exposed Infected Recovered (SEIR) model are discussed, such as SEIRD^21^ and the extended SEIRD^51^.

1. In^20^, the authors propose a joint framework for a collaborative city DT that employs Time Convolutional Networks (TCNs) and a federated learning solution. TCNs leverage causal and dilated convolutions to ensure robust sequence modeling and memory efficiency, utilizing temporal and historical data for infection prediction. Concurrently, an FL server iteratively updates and refines a global model centrally based on shared parameters from local models, ensuring privacy during infection prediction and management. The proposed architecture aids in understanding infection spread and outlines response strategies for effective infection management. Training occurs locally at each city-level DT, where the data resides, ensuring data privacy. Instead of sharing raw data, each local DT transmits model updates or parameters to the FL-based central server in a collaborative manner. This enables the FL-server to refine the global model and establish relationships between infection dynamics and management strategies, contributing to pandemic management at the city level. Experiments using real datasets from various U.S. States (i.e., COVID-19 Tracking Project Dataset and COVID-19 State Actions Dataset) demonstrate the effectiveness of the proposed solution. However, generalizing the model to cities with diverse infrastructures and socio-political contexts remains a challenge. The proposed solution is validated by its superiority over existing counterparts in pandemic forecasting, management, decision-support capabilities, and privacy protection.
2. The authors in ref. ^21^ employ a DT methodology to virtually represent three dynamic models illustrating the spread of COVID-19 in Catalonia. These models include: (1) a SEIRD-based System Dynamic (SD) model for preliminary system analysis, rapid prototyping, defining forecast boundaries, and testing assumptions; (2) a Python-based model optimized using a dual annealing algorithm to estimate transmission rates, containment coefficients, and reproduction numbers of the disease, enabling the evaluation of containment strategies and pandemic trend shifts; and (3) a Specification and Description Language (SDL) model employing a Cellular Automaton (CA) strategy for spatial analysis of infection spread, facilitating the adoption of preventive measures, advanced forecasting, and vaccination analysis across different health regions. The dynamic models are validated using a model comparison approach and a Verification and Validation (V&V) loop. The former refines model assumptions to ensure consistency among the behaviors of the SD, Python-based and SDL models, while the latter adjusts parameters daily by comparing real-world data (digital shadow) with simulation outputs. This process aids in understanding the impact of Non-Pharmaceutical Interventions (NPIs), such as lockdowns and mask distribution, in controlling the pandemic, and demonstrates predictions accuracy through comparisons with physical-world scenarios. For accurate assessment of the pandemic situation, the methodology highlights the importance of continuous validation and monitoring. The approach supports informed decision-making by understanding the causal relationships in pandemic dynamics and appropriately adjusting model assumptions for effective pandemic management. However, the methodology relies on datasets from different countries, which have inherent inconsistencies due to geospatial diversity, making its universal application a challenging task.
3. The author in ref. ^51^ have developed a DT model for predicting true infection trends and analyzing the effect of NPIs on the COVID-19 spread in Catalonia. The proposed model extends the SEIRD framework to the SCVEI_*R*_I_*D*_RHUD, encompassing cellular automata, compartmental modeling and optimization algorithms alongside vaccination dynamics, infection identification rates, and containment measures. Each cell of the CA represents a geographic region, with populations divided into compartments, each indicating a distinct state derived from the SEIRD model. A python-based optimization algorithm validates model parameters by comparing Catalonia Healthcare data (digital shadow) with simulated data (digital master) to enable pandemic forecasting and model validation. Model assumptions are defined using the SDL and implemented with Specification and Description Language Parallel Simulator (SDLPS) software, facilitating easy implementation and effective collaboration across disciplines. Validation is achieved through model comparison and solution validation mechanisms, with SDL ensuring continuous verification of assumptions against real-time data, while CA enables regional validations. Although, the model is calibrated using Catalonia-specific data, which limits generalization, the SDL-based design and modular approach make the model adaptable to diverse datasets and contexts, thereby enhancing its suitability for broader applications. The forecast model offers insights into infection dynamics, such as predicting saturation points or endemic trends, enabling decision-makers to monitor infections effectively and implement appropriate pandemic response measures across diverse regions.
4. The authors in ref. ^53^ propose a DT framework for pandemic simulation using healthcare and wastewater data to forecast and verify the spread of COVID-19 in Catalonia. Healthcare data pertains to symptomatic or tested individuals, whereas wastewater data provides insights from a broader population perspective, including asymptomatic cases. The proposed DT framework comprises a three-pronged model-based paradigm encompassing: a basic SEIRD-based system dynamic model for testing initial assumptions, developed in Insight Maker; an optimization model implemented in Python to capture variations in infection dynamics influenced by NPIs; and a graphical SDL model employing CA for regional predictions, developed in SDLPS software. The DT framework includes a continuous time-series-enabled weekly validation mechanism designed to correct prediction discrepancies between synthetic and real-world data (sewage and clinical) for enabling model recalibrations. Validation is further enhanced by relying on multiple data sources (i.e. sewage, clinical), particularly through the aggregation of wastewater samples from multiple treatment plants. This approach employs a wastewater-based validation system to address prediction inaccuracies caused by factors such as insufficient data, subclinical infections, variants, or behavioral changes in response to public health interventions, thereby enhancing accuracy and reliability of long-term COVID-19 spread predictions in society. Reliance on clinical and sewage data comes with numerous limitations as well. For instance, healthcare data reliability is affected by under-reporting or infrequent testing, while wastewater data reliability is affected by sampling errors and varying viral loads influenced by population density. Additionally, SDL model’s predictions are tailored to Catalonia-specific datasets, which may limit its performance when applied to regions with different infrastructure and data quality. The generalization of model assumptions (e.g., NPIs, vaccination rates, and variant dynamics) to other regions is not guaranteed.

### SDT for infections control (SDTIC)

*Scope:* A SDT for Infection Control may anticipate infection management strategies, such as quarantine, physical distancing, or organizing public health and awareness campaigns to promote vaccination plans and encourage preventive behaviors for regulating infection control.

*Approaches:* In this section, numerous SDTIC techniques are summarized, along with their evaluation methodologies, broader relevance, and challenges. These include some SEIR-based extension such as SEIR-V^23^ and the modified SEIR^52^.

1. In^22^, the authors present a navigation-based immersive system using DT and VR technology to visualize the role of interpersonal spacing in controlling the dissemination of SARS-CoV-2. The system uses Unity game engine to implement Social Distancing Algorithm in Virtual Reality (SoDAlVR). For this purpose, the ground floor of Markeaton St. campus at the University of Derby is visualized in an immersive 3D environment to study social distancing under crowd dynamics. The simulated environment contains numerous agents, each following one of the eleven navigation patterns to reach their goal waypoints using a graph-based waypoint navigation system tailored to Dijkstra’s algorithm. Upon arriving at a goal waypoint, each agent progresses to a new state in a state machine model. The agents are clearly differentiated based on their speed and goals. Using VR controllers, a user navigates the simulated crowded environment and gets warning via a Spatial UI element for breaking social distancing rules. This provides real-time visual feedback, validating the system’s functionality. The approach enforces the social distancing policy of maintaining a 2-meters distance by monitoring users in this multi-agent environment. Future plans include monitoring disease spread among agents and extending the system from VR to AR paradigm. However, the current simulation is limited to a specific use-case scenario of the University of Derby, predefined navigation patterns of agents, and the absence of epidemiological data for modeling infection spread patterns. Therefore customizations are required to generalize the system for other environments and applications.
2. In^23^, a novel statistical extension of the SEIR model is proposed for simulating and analyzing voluntary vaccination strategies during the COVID-19 spread. This model may be considered for possible inclusion into DT models. The proposed model is a two-layer graph-based SEIR-V model where the contact layer represents individuals (as graph nodes) and their contacts (as weighted edges), with the weight indicating a susceptible individual’s affinity for being infected after contact with an infectious individual. Additionally, the information layer represents the medium for receiving knowledge about the virus, either locally (through personal interaction) or globally (through mass media). Furthermore, each node employs a game-theoretic approach to make voluntary vaccination decision based on a cost-benefit analysis, aiming to maximize gains (e.g., protection from infection) minimize losses (e.g., vaccine side affects). The outcome of repeated Monte-Carlo simulations show that vaccination decisions based on global information lead to crowding and overloading of healthcare systems, while decisions based on local information result in evenly distributed vaccination efforts and reduced infection peaks. Additionally, prioritizing elderly population decreases mortality but increases infection rates, while prioritizing youth leads to the opposite effect. This research may provide valuable insights for policymakers in designing effective vaccination campaigns to control pandemics. The proposed model is evaluated with real COVID-19 data from Belgrade and exhibits satisfactory accuracy under the initial assumptions. However, relying solely on local data and not accounting for inter-city travel dynamics, health policy variability, and individuals at higher vaccination risks (e.g., pregnant women, chemotherapy patients) limits the model’s applicability to other regions and its generalizability to broader population.
3. The authors in ref. ^48^ develop an agent-based city DT framework to assess the socio-economic and public health impact of NPIs in managing the COVID-19 pandemic. The framework employs an agent-based modeling simulator trained on real-time data from city administration, incorporating details about individuals, locations, movement patterns, infection attributes, social preventive measures, and protective strategies to manage outbreaks. This enables the city DT to robustly forecast infection spread, predict features of affected individuals, and estimate burdens on healthcare and quarantine centers. The DT framework employs an iterative human-in-the-loop simulation strategy. It uses ESL based agent/actor technology to conduct simulations, which are interpreted using human decision-making and reinforcement learning-based what-if analysis. This approach allows the simulator to identify optimal intervention strategy for effectively managing the pandemic, thereby restoring social well-being, health, and the economy. The validity of the model assumptions is ensured by local demographic and epidemiology experts. The simulated results are validated by iteratively comparing them with real-time epidemic data provided by city authorities to ensure predictive accuracy and model convergence. The system holds promise for aiding pandemic-related decision-making. However, reliance on real-world data for validation presents challenges due to the limited availability of high-fidelity socio-demographic data. The proposed model evaluates the effectiveness of preventive measures in controlling infection and predicts the impact of protective measures. However, inaccuracies in model outputs may arise from oversimplified assumptions. Contextualizing the framework for Pune city limits its generalizability to other cities without significant adjustments. Finally, scaling the ESL technology infrastructure for state or national level implementation is computationally infeasible under the current scenario.
4. In^49^, the authors introduced a DT framework for infection control and management, focusing on optimizing large-scale COVID-19 vaccination centers. The framework aims to efficiently vaccinate a large number of individuals while minimizing time and resource utilization, particularly healthcare staff. This is achieved through a combination of a mobile application and a discrete event simulator. Clinical operators use smartphones equipped with Near Field Communication (NFC) readers to scan NFC badges of visitors, measuring time-stamped data on individual flow and phase durations during the vaccination process. The mobile app relays this data (e.g., queue lengths, resource utilization) for analysis and visualization via a dynamic dashboard designed for policy makers. The time-stamped data is analyzed at the DT, where a discrete event simulator processes around 100 time-stamped data points for each phase of the vaccination process. A data-fitting software is used for statistical distribution fitting (triangular, gamma, and Weibull) on the incoming data points. The validity of the model is ensured by measuring realistic variations in phase durations and through beta-testing in a smaller clinic. Using model parameters, the discrete event simulator models the vaccination process under various scenarios to optimize patient flow and address potential bottlenecks. The DT framework facilitates real-time data collection, analysis, performance tracking and improvement to enhance operational efficiency in vaccination centers. However, the framework has some limitations, including the exclusion of initial data points during the operator learning curve to maintain system accuracy. Additionally, being designed for specific walk-in clinic configurations reduces its immediate applicability to alternate settings.
5. In^50^, the authors created a virtual reality-based DT for remotely observing people and ensuring compliance with social distancing in a laboratory space. The DT was built using the Unity game engine and ProBuilder modeling tool, while laboratory occupants were detected using the YOLOv3 model trained on a dataset containing 2022 labeled images of individuals. The approach utilized the PyTorch KeypointRCNN model, with a ResNet50 backbone to estimate human body positions by calculating key joint angles and pairwise Euclidean distances, ensuring compliance with social distancing policies. The authors validated their approach using real-time images captured from the laboratory and synthetic images generated using VR. Although synthetic data is helpful for model training, it may not fully replicate the environmental variability of the real-world. Therefore, relying on a specific dataset may reduce the model’s generalizability and prediction effectiveness in diverse real-life scenarios. The results showed high accuracy in detecting people and predicting body postures. Specifically, a person-detection accuracy of 91% and 94% was observed for real and synthetic images, respectively. Whereas posture estimation achieved accuracies of 83.82% for real data and 84.73% for synthetic data. Furthermore, the correlation coefficient for Euclidean distances between real and synthetic images was 0.82, demonstrating a strong alignment.
6. In^52^, the authors employ an extension of the SEIR model to discuss the potential of DT in mitigating the spread of COVID-19. The modified SEIR model considers other population-specific parameters, such as the basic reproduction number (*R*_*o*_), average incubation duration (*Y*), and recovery duration (*D*), to predict infection spread trends during the 3rd wave of COVID-19 in Pakistan. The framework suggests that reducing social contact and enforcing social distancing can significantly decrease the spread of infection. Furthermore, DT models can predict infection spread while addressing four levels of infection management, enabling policymakers to plan effectively for lockdown measures and infection control. The validity of the framework is demonstrated by simulating infection spread under strict, partial, and no lockdown scenarios during the 3rd wave of COVID-19. The predictions align with observed trends and suggest that a gradual lifting of lockdowns is an appropriate strategy for effectively managing the disease spread. The model is based on a dataset from Pakistan, therefore generalizing it to other demographic regions with diverse socio-economic and political conditions may pose challenges. Additionally, since the model relies on the timeliness of officially released data, its predictions may deviate from real-world values if the data is not updated promptly with actual infection records.
7. In^54^, the authors present a collaborative framework integrating blockchain, DTs, and AI to establish secure medical cyber physical systems. The proposed framework aims to issue decentralized epidemic alerts for predicted risks and provide decision support to authorities by suggesting preventive measures such as quarantine, lockdown, and limiting social interactions to mitigate disease spread. The decentralized conceptual framework comprises four layers: (1) the physical layer–representing network nodes (i.e., humans and devices) contributing system data; (2) the blockchain-enabled DT layer–facilitating secure, distributed communication via a ledger among DTs (virtual network nodes); (3) the data analytics layer–featuring a ledger-oriented predictive model employing AI/ML techniques, providing offline and online predictions to support decision making; and (4) the decision-making layer–using consensus-driven coordination to issue alerts for managing pandemics effectively. Blockchain technology ensures distributed storage, direct-node communication, data integrity, and openness. The ledger-based predictive model integrates a database to record historical communication between DTs. In the offline phase, stored ledger information trains predictive models, which are refined through K-fold cross-validation and hyperparameter optimization, and validated with performance metrics like accuracy, precision, recall, and F1-score. The online model processes live streaming data for outbreak predictions and proposes decision strategies for pandemic containment. However, relying heavily on historical and live data may reduce prediction performance in cases of incomplete or biased datasets. Additionally, ensuring seamless connectivity and compatibility among nodes can be challenging. Generalizing the proposed framework to diseases beyond COVID-19 may require significant reconfigurations.
8. The authors in ref. ^55^ discussed a DT of Singapore General Hospital (SGH) developed for visualizing health data in a virtual environment. The conceptual 3-Dimensional Disease Outbreak Surveillance System (3D-DOSS) is designed to identify and respond to infectious diseases, focusing on the integration of medical information into a virtual hospital model to support effective disease monitoring and healthcare management. To create the virtual DT environment, the 3D-DOSS system employs the Unity game engine for spatiotemporal virtual mapping of SGH, AutoCAD for generating detailed floor plan templates, and SAP patient management data for spatial analysis of patients movements. By tracking patients, the DT system facilitates contact tracing, outbreak mapping, and cluster detection within the hospital. The validity of the system was demonstrated through its ability to outperform traditional methods in surveillance, contact tracing, and outbreak mapping. For instance, it traced inpatient COVID-19 exposures linked to an infected healthcare worker in April 2021, mapped OXA-48 outbreaks in 2020, and identified influenza (2018) and klebsiella pneumoniae (2018 and 2019) clusters among hospitalized patients. However, the 3D-DOSS system has certain limitations, including its reliance on manual data input, the absence of real-time data flows, and the unavailability of discharged patient information, which are critical factors in designing a robust disease surveillance system. Furthermore, its customization to SGH-specific infrastructure and data, along with the unavailability of predictive modeling features, limits the generalizability of this proof-of-concept prototype for outbreak detection and response in hospitals with different operations frameworks.

### SDT for infection combat (SDTICom)

*Scope:* A SDT for Infection Combat may predict preliminary methodologies for actively engaging with infections, such as medical diagnostics, antiviral/antibacterial drug development, and understanding host immune responses to directly confront and defeat infections.

*Approaches:* This section discusses numerous SDTICom techniques, along with their verification approaches, generalizability, and constraints.

1. In^24^, the authors introduced a DT prototype for human immune system to address various diseases. The article highlights that developing such a system poses significant challenges due to the intricacy of the human immune system and the issues surrounding data availability in vivo. However, the authors proposed a four-step strategy to address these challenges, which includes goal setting, adopting tailored approaches, thorough validation, and iterative enhancements. Goal setting involves specifying a use case, such as treatment forecasting or drug development, and constructing a general immune DT model template around it. A tailored approach involves customizing the general template with personalized data to address a patient’s specific scenario. Validation ensures thorough testing of model outcomes to address forecast uncertainties. Iterative enhancement signifies continuous refinement to improve model performance over time. Although no particular model or AI technique is presented for the anticipated immune DT, the paper emphasize the potential of hybrid approaches combining mechanistic, AI, ML and data-driven models for effective predictions. Additionally, platforms such as Cell Collective may be used for collaborative modeling, simulation and validation. Due to uniqueness of immune responses, generalizing such DTs build for one immune application to others would be a challenging task. However, extending common elements from a core model may expedite the development of specific immune applications. The authors emphasize the need for model and dataset repositories, however no particular datasets are discussed.
2. The authors in ref. ^25^ presented a DT blueprint modeling the human immune system at the cellular level to simulate immune defense mechanisms against infections. The blueprint comprises 27 immune cells (both innate and adaptive), 31 cytokines and immunoglobulins, and 9 distinct pathogens. It highlights cellular components and interactions among cells, molecules and pathogens, achieved through a comprehensive Systems Biology Graphical Notation (SBGN) map and multi-scale logical model. The immune model employs a rule-based methodology where biological components are discretized to depict ongoing activity, with logical rules characterizing interactions or state changes among them. The model is visualized using a layered SBGN map created in the CellDesigner editor, and uses the Minerva platform to support visualizations. The map organizes immune components into five levels, i.e., pathogens, non-immune cells, innate immune cells, adaptive immune cells, and secreted molecules. The validity of the model is ensured through its predictions of immune response to pathogens, as confirmed by experimental data, and its ability to simulate complex scenario, such as coinfections. To promote community-based validation, the proposed blueprint is openly accessible on the Cell Collective platform for further experiments and enhancements. The generalizability of the model is supported by its online availability, facilitating broader research and interdisciplinary coordination. However, its reliance on existing knowledge, the exclusion of certain immune components and cell movements, and its qualitative nature restrict its ability to capture the exhaustive immune response dynamics. Nevertheless, the proposed immune framework constitutes a remarkable contribution to the advancement of a comprehensive immune DT and fostering precision medicine.

### SDT for infection recovery (SDTIR)

*Scope:* A SDT for Infection Recovery may forecast recuperation and rehabilitation mechanisms, including effective planning, monitoring, and response systems, to restore societal norms following the containment or suppression of infections.

*Approaches:* Upon scrutinizing this field of research, a shortage of methodologies is evident. Innovative approaches are needed to address this research deficiency.

## Discussion

The proposed Rehan’s taxonomy of *societal digital twin* provides a novel framework for classifying digital twin strategies for infection containment across various stages of infection management, including initiation, spread, control, combat, and recovery. Based on the presented classification, SDT approaches targeting infection initiation and recovery remain largely unexplored, necessitating further research to bridge this gap. While limited strategies exist for infection combat, extensive investigation is necessary to develop robust immune response models and long-term recovery frameworks.

In contrast, several DT approaches have been developed for infection spread and control, including SEIR-based epidemiological models, TCNs and FL-based approaches, VR-based simulations, agent-based models, and blockchain-based DT implementations. While these approaches exhibit higher validation maturity, they often rely on region-specific datasets (e.g., Catalonia, Pakistan, Pune, Singapore), lack real-world environmental variability, or address highly specific use cases–such as visualizing the ground floor of Markeaton St. campus at the University of Derby or customizing SDTs for specific hospital infrastructures (Singapore General Hospital). Such constraints hinder the universal applicability of SDTs, emphasizing the need for scalable and adaptable implementations in diverse healthcare environments.

SDT models are data-driven AI/ML systems, requiring sophisticated infrastructure for data storage, processing, model training, and communication. Once trained, an SDT model necessitates continuous updates to mitigate algorithmic biases, ensure fairness, and maintain adaptability to evolving healthcare landscapes. However, healthcare dynamics vary significantly across regions, making cross-border validation essential to achieve both local accuracy and global scalability.

While current SDT strategies provide significant support for predictive modeling and healthcare decision-making, further advancements are necessary to enable personalized treatments, interdisciplinary adaptability, and seamless collaboration. Developing continuous validation frameworks, scalable SDT architectures, and universally adaptable solutions are imperative to establishing robust, flexible, and globally deployable SDT ecosystems for the future of healthcare.

## Limitations of societal digital twin

While applying SDTs has the potential to revolutionize the healthcare sector, however numerous limitations of SDT technology exist, including:

1. *Infrastructure Requirements:* Applying DT technology is an ongoing challenge that requires sophisticated sensor networks, secure communication protocols, high computational power, secure data storage, and advanced modeling, prediction and simulation techniques. As an advanced version of DTs, SDTs necessitate even greater infrastructure to process DT knowledge and efficiently predict infection-related decisions.
2. *Technological Co-existence:* SDTs encompass numerous technologies that are required to coordinate and collaborate in real-time to achieve the common goal of pandemic containment. Maintaining seamless communication demands compatibility, co-existence and interoperability among multiple technologies, which is critical for the successful realization of SDTs in healthcare.
3. *Secure Communication and Storage:* Transmitting patient data over a network is vulnerable to numerous challenges, such as eavesdropping, man-in-the-middle attacks, and denial of service attacks. SDTs are required to deploy robust safety mechanisms, such as encryption, authentication, firewalls, and intrusion detection systems. Likewise, secure storage can be ensured through encryption at rest, authentication, regular backups, and vulnerability management to address threats like ransomware, SQL injection, and malware.
4. *Performance Efficiency:* Efficient execution of tasks under throughput and response time constraints is indispensable for real-time communication in SDTs. It requires optimizing resource usage (e.g., computational power, storage, and network bandwidth) and implementing load-balancing mechanisms to prevent performance bottlenecks–a particularly challenging task in the highly data-centric SDT environment.
5. *Data Integration and Interoperability:* SDTs rely on clean and standardized data for optimal functioning. However, healthcare data is often fragmented across disparate systems and originates from diverse sources (e.g., sensors, MIoT, CT scans, and MRI, etc.). The inconsistent and fragmented nature of this data frequently renders it unsuitable for direct processing, posing significant challenges in SDT environments.
6. *Fairness and Dynamic Adaptability:* SDTs rely on AI/ML models for future predictions, which are trained on specific datasets. If these models are not properly trained, regularly updated, or if the datasets lack representative diversity or fail to adapt to emerging healthcare trends, prediction biases can occur. Such biases can severely impact the accuracy and reliability of SDT predictions. Therefore ensuring continuous retraining with the validated and unbiased data is a critical challenge to be properly addressed for maintaining accuracy and fairness in SDT-based predictions.
7. *Reliability:* Ensuring the stability and availability of SDT systems requires adopting mature technologies during implementation. It also involves ensuring that system operations can continue with reduced functionality (i.e., graceful degradation) in the event of hardware or software failures, as well as maintaining backups to enable recovery processes.
8. *Scalability:* Given the inherent complexities of SDT technologies and the vast volume of healthcare data, scaling SDTs across various societal levels (e.g., community, state, national, and global) introduces additional challenges, including cross-border standardization, interoperability with heterogeneous healthcare infrastructures, and the ability to integrate SDTs into multi-institutional frameworks for pandemic monitoring and response. These include managing interactions between SDTs and accommodating variations in healthcare regulations and ethical considerations, which can significantly influence infection management decisions.
9. *Adoption and Sustainability:* While DTs and SDTs represent emerging trends with enormous healthcare benefits, promoting these technologies as mature solutions requires widespread awareness campaigns. Additionally, the economic viability of SDTs demands substantial funding, posing a significant challenge to their long-term sustainability.

## SDT as a smart service—a smart, dynamic societal digital universe for pandemic containment

DT technology holds immense potential for improving personalized treatment, precision medical care, optimizing hospital resources, and facilitating drug R&D. However, creating a standalone DT is a highly complex task requiring specialized technical and medical expertise, advanced infrastructure (for data gathering, communication, networking, secure management, modeling and simulation), and seamless system management. These requirements make standalone DT deployment an expensive, time-consuming and arduous endeavor for independent entities (such as patients or physicians) and even for large organizations (such as clinics or hospitals).

To overcome the previously mentioned limitations, a viable solution is to adopt a service-oriented paradigm, where the service provider:

1. Manages all the underlying technical, data and infrastructure related complexities at the network core.
2. Ensures efficient processes for seamless bi-directional communication between users and the service backbone.
3. Provides on-the-fly and on-demand service availability to ensure real-time service and alert mechanisms.
4. Ensures scalability both vertically (enabling individual users to add or remove resources) and horizontally (to accommodate a growing number of users).
5. Innovates smartness by charging users based on various use case scenarios such as difference in the amount of uploaded and downloaded data, social status, demography, and so on.

In brief, the SDT as a Smart Service (SDTaaSS) paradigm incentivizes users who share valuable data through a subscription-based model, creating an adaptive and mutually beneficial healthcare ecosystem. This service-oriented approach enables patients, physicians, clinics, and hospitals to seamlessly input healthcare records into the system and receive real-time predictions, fostering greater adoption. Such a flexible, cost-effective, and efficient solution may foster an ecosystem for a *smart, dynamic societal digital universe*. The extensive health benefits of SDTaaSS could encourage customers worldwide to subscribe for the promotion of their well-being, making SDTaaSS adoption location-agnostic. Ultimately, it would enable the collection of global health data for efficiently predicting and managing pandemics such as SARS-CoV-2 (COVID-19) in the future.

### SDT: use cases and actionable insights

The deadly impact of COVID-19 across the globe has highlighted the urgent need to better prepare for any future healthcare calamity. In this context, SDTaaSS has substantial potential for analyzing, predicting and managing healthcare challenges in future. Below, a brief discussion of various use cases and actionable insights is presented to address future pandemics.

1. *Standalone Healthcare Entities:*
  - *Use Cases:*
    *Individuals:* are the data sources using wearables such as smart watches, pulse oximeters and heart pacemaker. These devices automatically collect data regarding temperature, blood oxygen level and heart rate and relay it to individual’s DT. Patients may manually input data (e.g. measurements from self-test kits for COVID-19 or daily water intake) into DT-enabled mobile apps.
    *Physicians:* input patient health records through interactive DT dashboards after identifying symptoms (e.g., cough, fever, sore throat) and performing diagnostic tests (e.g., blood tests for bacterial infections or PCR tests for viral infections like COVID-19 or influenza).
  - *Actionable Insights:* Once relevant information is collected, SDTaaSS can generate health predictions that are accessible to patients (via basic graphs) or physicians (via sophisticated graphs). In critical situation, the system may issue safety alerts to physicians or emergency department for taking timely measures to ensure patient well-being.
2. *Large Healthcare Entities:*
  - *Use Cases:*
    *Clinics and Hospitals:* may use interactive DT GUIs, allowing hospital staff to enter patient symptoms and admission details during normal operations and pandemic outbreaks. Furthermore, surveillance cameras at the hospital entry and exit points may send patient influx information to the DT via wired and wireless networks.
  - *Actionable Insights:* SDTaaSS may predict the severity of healthcare emergencies based on reported cases and visitor information. During pandemics, it can predict ICU occupancy, ventilators shortage, and issue alerts for staff scheduling and resource management. Additionally, public health authorities can receive alerts about infection hotspots to implement precautionary measures.
3. *Healthcare Policy-Making Entities:*
  - *Use Cases:*
    *Public Health Authorities:* monitor ongoing healthcare situations across community, state, national, and global levels through electronic, print, and social media. The interactive SDT GUIs and prediction forecasts assist them to make swift decisions during pandemic outbreaks.
  - *Actionable Insights:* Under the direction of public health authorities, SDTaaSS may issue alerts for smart lockdowns, travel quarantine monitoring, infection contact tracing, and vaccination and hygiene awareness campaigns to contain pandemics.

## Future challenges and research directions

DT technology is still in its early stages within the healthcare domain. There is a significant journey ahead to develop fully functional personalized, resource management, and societal DTs tailored to human healthcare needs. Addressing this task involves dealing with a myriad of issues, challenges, and research directions that require further brainstorming and exploration. Outlined below are the key areas of healthcare-related DT that demand significant investigation:

### Prognostic analytics and modeling

Forecasting the progression of medical conditions is crucial for effective disease management. However, effective prediction requires careful modeling of disease development patterns. It requires representative historical and ongoing health data, alongside behavioral, environmental, and biological factors, such as genomics, transcriptomics, proteomics, and metabolomics. Additionally, effective forecasting requires iterative tuning of model parameters to minimize the gap between model predictions and real-world observations.

Dynamic predictive models may anticipate health issues and disease development trends, thereby enabling informed decisions regarding prevention and cure methodologies in advance. Likewise, personalized physiological models can estimate future behaviors derived from collected data. However, implementing these models to replicate the intricate functionality and interactions of human organs in a DT environment presents a formidable challenge, requiring the refinement of AI-driven SDT models to enhance their adaptability to real-time epidemiological data. Addressing this challenge requires the development of robust mechanisms for constructing and refining dynamic predictive models. Achieving this goal entails thorough investigation and innovative solutions.

### Personalized treatment

The seamless availability of DT can provide an interactive environment. It may enable users to get insights into their personalized health conditions and suggestions to improve their well-being. However, developing personalized DT for predicting medications based on a patient’s age, health condition, comorbidities, and the genetic makeup (mutations and biomarkers) is a complex task that requires further investigation. A future challenge is to utilize machine and deep learning approaches to understand personalized patient profiles based on biological signatures and clinical phenotypes. It may enable physicians to prescribe personalized treatment and precision healthcare to patients.

### Personalized training

Medical treatments usually follow an established set of procedures and protocols to ensure safe treatment. A DT modeling and simulation environment can provide an ideal solution to implement complex medical procedures optimally. In doing so, it may provide an interactive, hands-on, and evidence-based training environment to medical professionals, where trainees may get 24/7 access to virtual medical resources, enhancing their competence and interdisciplinary knowledge. Moreover, DT may provide a flexible environment for skill evaluation by incorporating rigorous assessment, appraisal, and gauging procedures. A DT may serve as a resilient resource even in emergency conditions, allowing paramedic staff to receive fast-paced 24/7 training for dealing with critical situations or pandemics.

Despite the numerous advantages of DT technology in leveraging medical training, it may suffer from critical challenges as well. For example, modeling evidence-based training and adaptability mechanisms based on cutting-edge research and best practices in a DT environment is very challenging. Furthermore, complying with legal, ethical, and safety standards, along with cultural sensitivity issues, to leverage effective personalized training through DT is a future challenge. Therefore, there is still a long way to go to create personalized DT trainers that can provide professional-grade training comparable to that of experienced healthcare professionals for effectively handling crisis situations.

### Interdisciplinary coordination

As a multidisciplinary field, SDT requires effective communication among stakeholders (e.g., patients, data scientists, bioinformatics specialists, and healthcare professionals) to ensure seamless development, integration, and innovation in the healthcare domain. Facilitating such collaboration promotes actionable insights and knowledge transfer among stakeholders, which is crucial for effective SDT implementation.

Furthermore, standardizing communication protocols can streamline structured data sharing and role-based access control, both of which are essential for fostering a collaborative environment that integrates diverse expertise from healthcare and technology. Therefore, modeling a versatile interdisciplinary SDT framework in the healthcare domain, where all stakeholders achieve a win-win scenario, remains a significant challenge.

Key actionable steps for interdisciplinary coordination among experts from data science, medicine, and engineering include:

1. *Shared Goals:* Define actionable goals aligned with the priorities and objectives of relevant stakeholders.
2. *Standardized Communication Protocols:* Develop protocols to mitigate communication gaps and foster a culture of diversity and collaboration.
3. *Advanced Healthcare Platforms:* Leverage cutting-edge technologies such as AI, ML, AR, VR, ER, DT, and blockchain to create advanced healthcare metaverse platforms for seamless collaboration in the digital world.
4. *Training and Workshops:* Organize interdisciplinary sessions to equip stakeholders with essential interdisciplinary knowledge and expertise.
5. *Feedback Mechanism:* Establish regular channels to highlight and coordinate unfinished tasks, improving workflow efficiency and progress tracking.
6. *Appointing Facilitators:* Consult interdisciplinary leaders to resolve communication gaps by streamlining shared workflows and fostering team cohesion.
7. *Key Performance Indicators (KPIs) Metrics:* Utilize KPIs to evaluate interdisciplinary coordination and refine objectives and strategies accordingly.

### User experience

For effective human-computer interaction, a DT may embody properties such as responsive web design, fast loading web apps, easy access and navigation, personalized dashboards, browser compatibility, and customer feedback mechanisms. Moreover, user experience can be improved by creating intuitive user interfaces and informative graphs. This may aid in an increased understanding of data. It may empower patients, technologists, and healthcare professionals to make more informed decisions about ongoing medical conditions. However, devising effective mechanisms for capturing and understanding user preferences and leveraging them to enhance user experience in the digital arena is a significant challenge.

### Digital twin availability

Like many other new and demanding technologies, initial DT products are likely to be costly, considering general market trends. Due to financial disparities, life-changing and life-saving DT technology may only be accessible to wealthy patients, thereby promoting social inequality and unfairness^32^ and further exacerbating socio-economic disparities^29,32^. Henceforth, it is imperative for governments, non-profit organizations, and health insurance companies to invest in the R&D of DT technology. Doing so may help make DT technology available to the general population. Additionally, it may potentially mitigate the trend of healthcare technology usage being contingent on social status and economic well-being.

### Digital twin scalability

DT ranks among the most demanding technologies of the information era, which is presumed to be readily adopted by future healthcare systems. Achieving this requires the development of expandable and adaptable approaches for creating more robust and flexible DT solutions in the future. A scalable DT may scale up horizontally or vertically, thereby aligning with the Digital Twin as a Service (DTaaS) paradigm. In the case of horizontal expansion, DTaaS may facilitate the emergence of new DT(s) within the DT ecosystem, whereas vertical expansion refers to maturing the existing DT(s) by adding new functionality under the DTaaS mechanism. In any case, scalability would increase the amount of data received, processed, and analyzed by the DT ecosystem.

However, ensuring efficiency while accommodating increasing user requirements is paramount for implementing scalable DT architecture in the medical domain^34^. Achieving this entails rigorous research into futuristic DT systems capable of accommodating the growing functionality and demand of society at both local and global levels, while addressing corresponding scalability-related bottlenecks. However, enhancing scalability would introduce complexities in DT systems, necessitating more robust solutions for data storage, processing, networking, and communication. By proactively planning and addressing such challenges, data scalability issues may be properly addressed, and the optimized performance of SDTs may be ensured in the future.

### Digital twin—a decision support system

Information and Communication Technologies (ICTs) have been pivotal in the *digitization and automation of industries*, including healthcare. Thanks to ICTs for significantly contributing to materializing the concept of *social distancing* during the COVID-19 outbreak, thereby saving millions of lives during the pandemic. In the prevailing scenario, there may be variability between the outcomes of DTs and real-world healthcare systems. Therefore, ICTs still have a long way to go in imitating complex natural processes of the human body and realizing the idea of a fully functional *humanoid DT*^7^.

Since DT technology is in its early stages of adoption within the healthcare sector, it may be considered more suitable for decision support rather than decision making^29^. Establishing trust in the decision-making capabilities of DTs depends on their ability to accurately predict early disease diagnosis and suggest preventive measures accordingly. Closing the disparity between DTs and physical-world healthcare systems is imperative to optimize the capabilities of DTs in enhancing medical decision-making and represents a significant research challenge to tackle.

### Intelligent digital twin (IDT)—hospital process optimization ecosystem

By conducting predictive analyses, a DT can serve as a valuable resource for streamlining hospital processes, such as patient influx management, staff scheduling, medical equipment maintenance, parking allocation, and building infrastructure adaptation. However, within the evolving landscape of digital healthcare, there is a growing demand for an IDT ecosystem. Such an ecosystem can comprehensively address the complex challenges of process optimization in the healthcare domain, offering *end-to-end solutions* for these critical areas.

The proposed *IDT architecture* may comprise four layers of DTs to ensure hospital process optimization. At the grassroots Layer-1, departmental DTs may exist to execute basic departmental-specific processes within a hospital. Building upon this foundation, Layer-2 may consist of inter-departmental DTs, focusing on process optimization and seamless coordination across various departments in a hospital environment. Layer-3 may include inter-domain DTs, facilitating cross-domain process optimization, such as forwarding an inventory shortage request from a hospital to the corresponding supplier’s domain. Finally at Layer-4, a central core DT may orchestrate end-to-end problem-solving and collaboration among various hospitals to achieve healthcare optimization across regions, countries, and continents globally.

The central hub of the IDT may critically analyze data, identify bottlenecks, and disseminate optimized solutions to bottlenecked DTs through a feedback-looping mechanism. Such a DT clustering approach can empower human experts to make informed decisions and tackle worldwide societal issues, including starvation, water crises, climate change, and so on. However, unlocking the full potential of IDTs demands extensive investigation to overcome the complexities of such a sophisticated architecture. By harnessing advanced technologies and fostering interdisciplinary collaboration, the IDT ecosystem has the capacity to revolutionize service delivery, enhance patient care, and address complex healthcare challenges on a global scale.

### Digital twin—development cost and time

A healthcare DT system requires a combination of various technologies and skill sets. Designing such a system necessitates healthcare domain knowledge as well as technological expertise in various areas such as big data analytics, machine/deep learning, AI, IoT, cloud/edge computing, database management, data visualization, and VR/AR/Mixed Reality (MR). Developing DTs using diverse technologies and skill sets may result in robust system design and development.

However, the downside is increased cost, complexity, and time consumption. Therefore, it is the key responsibility of the DT designer to proactively assess the aims and outcomes of the DT model, so that a DT can be devised using minimal efforts and resources. Building robust DT systems with minimal cost and time is another research challenge pertaining to DT system architecture.

### Ethical considerations

Ethical considerations in healthcare demand transparency and respect for patient autonomy within the DT environment. Transparent communication about the purpose of data usage and storage fosters trust between patients and healthcare providers, encouraging and motivating patients to share information confidently. A DT system may ensure patient autonomy^34^ by empowering patients with control over their data, including the ability to manage and revoke consent at any time. Furthermore, a DT may facilitate informed consent^34^ by notifying patients about the usage, storage, and analysis of their data while emphasizing the associated benefits for their well-being.

Additionally, informing patients about high standards of data privacy – such as encryption (e.g., Advanced Encryption Standard^56^, Homomorphic Encryption^47^, Elliptic Curve Cryptography^57^), access control (e.g., Role Based Access Control^58^, Attribute Based Access Control^59^), and anonymization (e.g., Differential Privacy^60^, l-diversity^61^), – is crucial for achieving customer satisfaction. However, integrating all these requirements into a DT environment is a challenging task that requires further investigation.

Ethical considerations may also involve promoting demographic diversity. This can be achieved by encouraging equitable representation and respecting cultural sensitivity in collecting, storing and examining data. Such practices may enable equal and easier participation of diverse groups, ensuring data authenticity, fairness and preventing biases. Moreover, maintaining a high level of data fairness in decision-making is essential. This can be achieved by training DT models on diverse, representative and balanced datasets^29^. However, achieving data fairness while considering demographic diversity poses a significant challenge and requires further investigation.

### Regulatory compliance

Regulatory compliance involves ensuring that DT technology complies with relevant laws, procedures, and standards for regulating patient data. The primary objective is to uphold high standards of security, integrity, confidentiality, privacy, availability, and protection of health-related data. Regulatory compliance fosters transparency in data usage and storage, guarantees patient rights, and promotes innovation^34^. It instills confidence among patients in sharing their personalized information readily, thereby streamlining data collection processes and enabling the availability of vast volumes of real-time healthcare information for conducting disease analysis and innovating treatment methodologies.

Currently, a multitude of laws governing healthcare-related data processing has been promulgated worldwide. These include the E.U.-based General Data Protection Regulation (GDPR)^29^, the U.S.-based Health Insurance Portability and Accountability Act (HIPAA)^62^, the Japan-based Act on the Protection of Personal Information (APPI)^63^, and the Australia-based Privacy Act 1988^64^. Additionally, regulations from governing bodies like the European Medicines Agency and the U.S. Food and Drug Administration (FDA) play a crucial role. Furthermore, the International Medical Device Regulators Forum^65^ is a key player in establishing common healthcare device-related regulatory standards to promote cross-border collaboration and healthcare innovation.

Given the growing influence of AI technology in the healthcare sector, the European Artificial Intelligence Act (AI Act)^66^ has recently been introduced to regulate the safe and sensible deployment of AI within healthcare applications. Likewise, the FDA is actively outlining guidelines for incorporating AI/ML technology into medical devices. Furthermore, standards for information security management systems, such as ISO 27001, exist and may serve as valuable resources for supporting future innovations in the healthcare sector.

To summarize, navigating regulatory compliance remains a complex challenge in the realm of healthcare-related DT. However, in the growing era of AI technology, there is an urgent need to expedite the drafting of new regulations (e.g., regarding data protection, dissemination, and patient approval^33^) to keep abreast of state-of-the-art innovations in healthcare-related R&D. It is also proposed to create an International Societal Digital Twin Regulatory Organization and Global Healthcare Metaverse Regulations Authority for drafting effective laws and procedures for resolving the regulatory issues among regions, bringing the stakeholders to one platform, and meeting the demands of healthcare-related Digital Twin technologies and the metaverse for welcoming futuristic healthcare innovations in the medical field.

## Emerging paradigms in societal digital twins

This section explores next-generation paradigms advancing SDTs toward proactive, intelligent, and human-centric systems across public health and future smart societies.

### Innovations in AI-aided digital twins

The convergence of Artificial Intelligence, cognitive systems, distributed learning, and human-centric modeling is redefining the capabilities of DTs. This evolution is producing intelligent, collaborative, and personalized systems that go far beyond traditional simulation. SDTs now operate across public health, education, mobility, and governance to anticipate needs, coordinate large-scale interventions, and support ethically aligned decision-making. In this section, we synthesize five key paradigms-Generative, Cognitive, Explainable, Federated, and Human-Centric Digital Twins-that are shaping the next generation of SDTs, supported by recent advancements in deep learning, affective computing, and edge-to-cloud orchestration.

• *Generative Digital Twins*: Generative Digital Twins are realized through deep generative models such as variational autoencoders, diffusion models, and generative adversarial networks, which enable the synthesis of high-dimensional, time-dependent, and realistic data. Unlike conventional simulation models, these systems learn from real-world data to reproduce and forecast complex phenomena. In healthcare, generative twins are already transforming clinical trials by simulating virtual control arms, patient trajectories, and counterfactual outcomes, thereby reducing the cost and time required for patient recruitment. In drug discovery, generative twins can replicate biological systems at multiple levels-from single cells to whole organs-and perform in silico perturbation experiments, supporting biomarker identification and toxicity prediction. These models operate bidirectionally: initialized with real patient or biological entity data, they generate predictions that feed back into decision-making loops. As computational capacity and biomedical datasets grow, generative Digital Twins are expected to underpin future large-scale, ethically compliant simulations in precision medicine and societal-scale public health forecasting^67^.

• *Cognitive Digital Twins:* Cognitive Digital Twins extend traditional digital twins by incorporating three intelligent layers: access, analytics, and cognition. These layers enable real-time data communication, embedded edge analytics, and complex decision-making capabilities. Unlike conventional twins, cognitive twins act as autonomous agents that interpret context, learn from interactions, and collaborate with other twins. In smart manufacturing, they can self-diagnose anomalies, correlate operational data with environmental and product-specific factors, and initiate adaptive actions or seek assistance from peer twins. This distributed intelligence enables predictive maintenance, process optimization, and cross-machine learning. Translated to public health, cognitive twins could autonomously assess evolving epidemiological trends, simulate intervention outcomes, and coordinate responses across digital health agents. Over time, their continuous learning feeds into a shared knowledge graph, enabling cumulative intelligence and resilience across societal systems^68^.

• *Explainable Digital Twins:* Explainable Digital Twins embed principles of interpretable and trustworthy machine learning to ensure transparency and auditability of AI-driven decisions. These systems use model-agnostic techniques and inherently interpretable algorithms to clarify how predictions are made and which features influence outcomes. This is critical in high-stakes domains such as healthcare and infrastructure, where explainability fosters trust and regulatory compliance. For example, in public health, explainable twins can justify risk forecasts for vulnerable populations during pandemics. In critical infrastructure, they support predictive maintenance by linking component degradation to specific sensor signals. In governance, they enhance accountability by clarifying why certain interventions are prioritized during emergencies^69^.

• *Federated Digital Twins:* Federated Digital Twins are evolving into interconnected networks that transcend isolated systems by forming a unified, cyber-physical fabric-referred to as the Internet of Federated Digital Twins (IoFDT). These systems enable real-time cooperation between diverse and distributed DTs through both horizontal (peer-level) and vertical (hierarchical) interactions. In public health, this allows the integration of lifestyle DTs with hospital systems for preventive care. In smart mobility, autonomous vehicle twins can interoperate with traffic and city infrastructure twins for predictive routing. IoFDTs ensure scalability, low-latency analytics, and context-aware coordination through AI-native networks and edge-cloud orchestration. This shift from isolated DTs to federated intelligence offers a resilient digital backbone for Society 5.0, enabling globally coordinated yet locally autonomous services^70^.

• *Human-Centric Digital Twins:* These extend traditional digital twin frameworks by integrating physiological, psychological, and cognitive models to create personalized, adaptive digital replicas of humans. These twins are particularly valuable in smart societal systems where human well-being, learning, and participation are prioritized. In healthcare, Human-Centric Digital Twins (HCDTs) can assist in real-time ergonomic monitoring and mental health support through emotion-aware modeling. In education, they can adapt teaching pace and content to individual cognitive traits and learning preferences. In workforce development, HCDTs support up-skilling and safe task delegation through dynamic capability modeling. Enabled by real-time sensing, bidirectional feedback, and knowledge-sharing frameworks, HCDTs bridge physical and virtual spaces to enhance individual agency and systemic resilience^71^.

### Metaverse arena: a futuristic approach for designing healthcare metaverse as a smart service (HMaaSS)

A metaverse is a fully connected digital universe, encompassing immersive 3D visual graphics (or virtual projections) of real world spaces. Each virtual projection mimics the behavior of its corresponding physical-world object.

A user (represented as an avatar) may enter the fully immersive environment of a metaverse using VR, AR, or MR headsets. They can navigate the virtual world and interact immersively with virtual objects or participants at a 360-degree angle in a multi-sensory manner (e.g., using sight, sound, and sometimes touch via haptic feedback). The environment of a metaverse can be categorized as either static or dynamic, as follows:

• *Static Metaverse:* The environmental constructs of a static metaverse are rendered based on pre-designed visualizations. Therefore, users experience the same immersive environment during each entry. For example, virtual tours of a museum with fixed artifacts are a static metaverse application, thereby making it suitable for training purposes.

• *Dynamic Metaverse:* The environmental constructs of a dynamic metaverse are seamlessly updated based on real-time communication and the changing states of physical-world objects. Therefore, dynamic metaverse incorporates DT technology. For instance, watching a hockey, cricket or football match immersively in a virtual playground where avatars of players update their movements based on the real-time actions of physical-world players.

A *static healthcare metaverse* can serve as a cost-effective tool for conducting training sessions for healthcare staff, where users can observe procedures from a 360-degree perspective. On the other hand, a *dynamic healthcare metaverse* may enable a team of physicians to interact immersively, regardless of their physical location, for advanced healthcare delivery to patients. For example, physicians can wear AR, VR or MR headsets to inspect a 3D model of a patient’s heart (as a DT) from all angles in a virtual operation theater. They can also predict outcomes of procedures, such as operating on arteries or inserting stents, based on the prevailing health conditions of a patient, such as blood flow or oxygen levels in the physical world.

A *Societal Healthcare Metaverse (SHM)* can be an efficient tool for addressing infections at the societal level. For this purpose, a team of physicians, data scientist and decision makers from around the globe can meet in a spatially-independent, fully immersive SHM environment to share collaborative insights on managing infections and predicting their social impact. Using simulations within the SHM, physicians can brainstorm across geographical boundaries to analyze infection patterns at both local and global scales. In the metaverse settings, data scientists can provide predictive visualizations, and physicians can collaborate to suggest countermeasures. Based on these recommendations, decision-makers can implement quick action plans in real time.

**Fig. 8:**
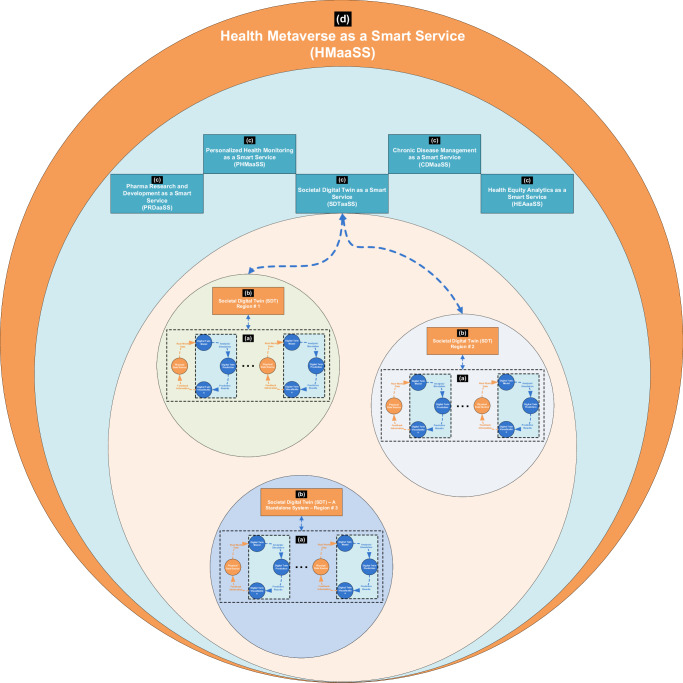
Conceptualization of the interaction among DT, SDT, SDTaaSS, and HMaaSS. This figure conceptualizes different levels of healthcare-related innovations supporting the implementation of DT technology in the healthcare sector. **a** Represents various DTs encompassing different regions (i.e., *Region#*1, *Region#*2, and *Region#*3). **b** Depicts multiple SDTs at the regional level. Here, the SDTs of *Region#*1 and *Region#*2 are governed by national, international, or global organizations based on a smart service model. Meanwhile, the SDT of *Region#3* operates as a standalone system, managed by a regional organization. **c** Highlights various smart services paradigms, including the SDTaaSS, which extends smart societal services for pandemic control to *Region#*1 and *Region#*2. **d** Showcases the HMaaSS, which is designed to empower communities at large by providing state-of-the-art healthcare services to enhance public well-being and foster a globally healthy society.

A fully functional *HMaaSS* would provide a smart, cost-effective, and flexible platform to deliver all healthcare-related services, including SDTaaSS, as a modular offering, as depicted in Fig. 8. This would enable customers to create customized, cost-effective, and on-demand solutions while avoiding implementation complexities. Furthermore, HMaaSS could drive public health innovation by facilitating novel healthcare solutions to protect humanity against deadly diseases, especially infectious ones like COVID-19, by integrating individual patient care with comprehensive societal strategies. It could serve as a valuable resource for supporting global health efforts (e.g., international vaccination campaigns, real-time pandemic data monitoring) and containing deadly pandemics like SARS-CoV-2 (COVID-19). By enabling real-time tracking and response to pandemic dynamics (such as infection initiation, spread, control, combat, and recovery), public health campaigns could be effectively organized to contain and manage infections.

Although there is a growing trend in DT development, the metaverse concept is still in its inception stages. Unlocking the full potential of healthcare metaverse requires substantial research to establish intelligent immersive environments that seamlessly integrate patient healthcare data between virtual and physical counterparts. However, implementing the metaverse demands specialized skill sets to efficiently integrate foundational technologies such as DT, IoT, AI/ML, 5G/6G, cloud/edge computing, VR, AR, MR, and blockchain. Due to limited expertise in metaverse integration, ensuring seamless connectivity, compatibility, co-existence, interoperability, and real-time synchronization among various technologies–without introducing millisecond-level delays–remains a significant challenge. Achieving such precise connectivity and compatibility is essential for realizing the true potential of the metaverse and presents an important area for future research.

## Conclusion

Digital twin technology presents enormous potential for revolutionizing the healthcare sector. This survey explores its role in infection containment and response by conceptualizing *societal digital twin* technology within a structured framework. This survey contributes in many ways. It conceptualizes SDT for infection control under a novel structured taxonomy (Rehan’s Taxonomy) that categorizes SDTs into five categories, namely infection initiation, spread, control, combat, and recovery. The proposed classification can help to manage pandemics through different infection response stages. By organizing numerous SDT approaches, this survey highlights their validation strategies, generalizability, and limitations, thereby underscoring state-of-the-art developments in various areas of SDT. Beyond classification, this survey examines various applications, data-driven design issues, key components, limitations, potential challenges, research opportunities, and evolving paradigms of DT technology in healthcare. Additionally, it introduces the smart service-oriented concepts of SDTaaSS and HMaaSS, which are expected to become viable soon, driven by the growing demand for DT technology in healthcare. The impact of these contributions is multi-fold. Its viability will be further supported by advancements in sensors, IoT, communication networks (5G/6G), AI/ML, data storage, cloud/edge computing, VR/AR/MR, and blockchain technologies. The availability of SDTaaSS and HMaaSS has the potential to empower communities, enhance societal well-being, and contribute to the creation of a healthier society and a promising future. It may help advance healthcare innovation and assist in effectively combating pandemics (such as COVID-19) across various levels of the social fabric, including local, communal, regional, and global levels. Ultimately, the adoption of SDTs in healthcare could drive transformative change, enabling data-driven decision-making, enhancing pandemic preparedness, and strengthening global resilience against future outbreaks.

## Data Availability

No datasets were generated or analysed during the current study.
